# Accuracy of Ionizing‐Radiation‐Based and Non‐Ionizing Imaging Assessments for the Diagnosis of Periodontitis: Systematic Review and Meta‐Analysis

**DOI:** 10.1111/jcpe.14137

**Published:** 2025-02-12

**Authors:** Nicola Discepoli, Isabella De Rubertis, Cecile Wasielewski, Giuseppe Troiano, Maria Clotilde Carra

**Affiliations:** ^1^ Department of Medical Biotechnologies, Unit of Periodontology Università degli Studi di Siena Siena Italy; ^2^ U.F.R. of Odontology Université Paris Cité Paris France; ^3^ Department of Clinical and Experimental Medicine University of Foggia Foggia Italy; ^4^ METHODS Team, CRESS, INSERM, INRAe Université Paris Cité Paris France; ^5^ Department of Translational Medicine University of Ferrara Ferrara Italy

**Keywords:** cone‐beam computed tomography, non‐ionizing imaging technique, periodontal diagnosis, periodontitis, radiography

## Abstract

**Aims:**

To evaluate the diagnostic accuracy of periapical, bitewing or panoramic radiographs (standard 2D radiographs) in detecting and monitoring periodontitis (PICO 1) and to assess the clinical relevance of alternative and emerging diagnostic methods (e.g., cone‐beam computed tomography [CBCT], magnetic resonance imaging [MRI], ultrasound imaging [USG]) compared to standard 2D radiographs or clinical/intra‐surgical examination in the diagnosis and surveillance of the disease (PICO 2).

**Materials and Methods:**

A systematic literature search was conducted through MEDLINE EMBASE, Scopus and Cochrane Library. When feasible (*n* > 2 comparable studies), a meta‐analysis of diagnostic accuracy was performed.

**Results:**

For PICO 1, 26 studies met the inclusion criteria. Pooled‐data analysis from three studies showed a sensitivity of 0.77 (95% confidence interval, CI: 0.66–0.85), specificity of 0.76 (95% CI: 0.64–0.84) and accuracy of 0.82, with a diagnostic odds ratio (DOR) of 137.99 (95% CI: 6.99–368.90). For PICO 2, 51 articles were included dealing with different techniques. The meta‐analysis for CBCT (three studies) showed a pooled sensitivity and specificity of 0.98 (95% CI: 0.96–1.00) and 0.98 (95% CI: 0.95–1.00), respectively, and a diagnostic accuracy of 0.99 in the detection of furcation involvement compared to intra‐surgical measurements.

**Conclusions:**

Standard 2D radiographs appear to have adequate diagnostic accuracy for periodontitis, while CBCT is highly sensitive and specific to detect and classify furcation involvement. The role of non‐ionizing techniques (MRI and USG) in diagnosing periodontitis remains under investigation.

## 1 Introduction

Periodontitis is a ubiquitous dysbiotic inflammatory disease mediated by bacterial dental biofilms (Caton et al. 2018; Sanz et al. 2020) and characterized by progressive destruction of the tooth‐supporting apparatus. At a patient level, according to the 2018 WWP Classification (Caton et al. 2018), the diagnosis of periodontitis is based on clinical and radiographic parameters (Tonetti et al. 2018). Two‐dimensional (2D) radiographs provide abundant information that cannot be obtained by any other non‐invasive method (e.g., root length, root form, periapical lesions, root proximity, estimates of density and amount of residual alveolar bone) and are an essential component of a complete periodontal examination (Armitage 1995). Periodontitis definitions, although based on marginal radiographic bone loss, suffer from severe limitations because they are not sensitive enough and may miss detection of mild to moderate periodontitis (Lang and Will 1977). Indeed, seminal in vitro experiments have indicated that the unaided eye could detect radiographic changes when approximately 50% of the bone mineral has already been lost (Ortman et al. 1982).

Classical literature on radiographic interpretation points out that radiographs usually underestimate the true amount of bone loss (Goldman et al. 1957; Theilade 1960) and extensive loss of clinical attachment can occur before there is radiographic evidence of bone remodelling (Goodson et al. 1984; Prichard 1961). At the site level, within the multidimensional staging process in use (Tonetti et al. 2018), radiography contributes to the assessment of specific key diagnostic factors such as infrabony defects and ridge defects, thus optimizing prognosis and individual treatment plans.

Two‐dimensional ionizing radiation techniques (Mallya and Lam 2018) suffer from inherent and well‐documented limitations such as distortion, superimposition and misrepresentation of structures (Kasaj and Willershausen 2007; Woelber et al. 2018). To overcome these limitations, 3D imaging techniques, such as cone‐beam computed tomography (CBCT), have been introduced. More recently, non‐ionizing 3D modalities (e.g., magnetic resonance imaging, MRI) have been proposed to improve diagnostic accuracy without exposing the patient to the risks associated with ionizing radiations.

Therefore, the present systematic review and meta‐analysis was conducted to answer the following research questions:

1. What is the diagnostic accuracy in detecting and monitoring periodontitis and periodontal changes over time of standard 2D radiographs (including periapical, bitewing and panoramic) taken in an adult patient seeking a dental diagnosis and/or treatment? (PICO 1).
2. In individuals assessed for periodontitis, what is the diagnostic accuracy and clinical relevance of alternative and emerging diagnostic methods (i.e., CBCT, MRI, subtraction radiography, periodontal endoscopy, optical coherence tomography, fluorescence spectroscopy, ultrasound imaging [USG], intraoral scanning, absorptiometry and xeroradiography) in detecting and monitoring the disease compared to standard 2D radiographs or clinical measurements? Specifically, do these methods provide more accurate, earlier, safer and cost‐effective diagnosis? (PICO 2)

Novel approaches, such as artificial intelligence technologies, are deliberately excluded from the present review.

## 2 Methods

### 2.1 Protocol Development and Research Questions

This review was registered at the PROSPERO International Prospective Register of Systematic Reviews (CRD42024574265). The systematic review protocol was structured and reported according to the PRISMA statement checklist (Moher et al. 2009).

### 2.2 Criteria Definition for the PICO Questions

#### 2.2.1 Population

Adults being assessed for periodontitis.

#### 2.2.2 Intervention (Index Diagnostic Test)

For PICO 1: standard 2D imaging modalities, including periapical, panoramic and bitewing radiographs. For PICO 2: alternative and emerging imaging and diagnostic methods including CBCT, MRI, subtraction radiography, periodontal endoscopy, optical coherence tomography (OCT), fluorescence spectroscopy, USG, intraoral scanning, absorptiometry and xeroradiography.

#### 2.2.3 Comparison (Reference Standard)

For PICO 1: clinical reference measures (including both clinical examination and intra‐surgical measurements) to obtain periodontal measurements (e.g., clinical attachment level [CAL]). For PICO 2: also, standard 2D radiographs.

#### 2.2.4 Outcome

Accuracy in the detection, staging, monitoring and surveillance of periodontitis or periodontal parameter changes over time.

### 2.3 Literature Search Strategy

Once the selection criteria have been determined, the following databases were screened by two independent reviewers (C.W. and I.D.R.): MEDLINE (through PubMed), EMBASE, Scopus and Cochrane Library. A grey literature search was conducted on OpenGrey and Google Scholar databases. A specific research equation was formulated for each PICO question and adapted to the different databases using keywords and MeshTerms. The literature search and selection process were conducted with the help of Ryyan software (https://www.rayyan.ai) in a stepwise manner, first upon title and abstract and then on full text. Any disagreement between the two reviewers was solved by consensus or eventually with discussion with a third author (M.C.C. or N.D.) acting as moderator. Concordance between the reviewers was assessed by estimating the kappa value and percentage of agreement.

### 2.4 Literature Search Restrictions

#### 2.4.1 Eligibility Criteria

- English literature.
- Human clinical studies in adult populations.
- The target condition being the disease (i.e., periodontitis), disease stage or a complicating condition associated with the clinical manifestation of the disease (e.g., intrabony defect).
- Periodontitis case definition based on epidemiological criteria (Page and Eke 2007) or clinical parameters, according to the 2018 WWP Classification or previous classification systems.
- A minimum sample size of 20 patients or 20 teeth/sites, according to the statistical units considered.
- Study designs: cross‐sectional, case–control, longitudinal (with a minimum follow‐up of 6 months) (Deeks et al. 2023), randomized controlled trials (RCTs). For retrospective studies, the time interval between the index test execution (e.g., CBCT) and the reference standard (e.g., clinical examination) was not to exceed 6 months.
- Owing to the expected heterogeneity in the literature, any measure of accuracy, validity, reproducibility or performance of the diagnostic test versus the reference standard(s) was considered.

### 2.5 Critical Evaluation

The critical evaluation was conducted by two reviewers (I.D.R. and C.W.) using the critical review checklist outlined in the revised Quality Assessment of Diagnostic Studies (QUADAS‐2) (Whiting et al. 2011). A consensus was reached through discussion in case of disagreement. Reporting bias and sponsoring bias were also evaluated.

### 2.6 Data Extraction

Two reviewers (M.C.C. and N.D.) performed an independent data extraction on a dedicated excel spreadsheet. A third reviewer compared the files, checked for possible discrepancies and compiled the final database that was used to produce summary tables for the qualitative and quantitative syntheses (meta‐analysis, whenever possible).

### 2.7 Data Analysis

The feasibility of a meta‐analysis was checked by the PICO question and the diagnostic technique used. Meta‐analysis was undertaken when sufficient comparable studies (*n* > 2) were available. If judged feasible, a meta‐analysis of diagnostic accuracy was conducted using the hierarchical summary receiver operating characteristic (HSROC) model (Rutter and Gatsonis 2001), accounting for both within‐study and between‐study variability. True positives (TPs), false positives (FPs), true negatives (TNs) and false negatives (FNs) were extracted from the individual studies and used to calculate sensitivity, specificity and accuracy.

Diagnostic odds ratios (DORs) were computed to summarize test performance across studies, and a random‐effects model was used to pool log‐transformed DORs. The pooled DOR, sensitivity and specificity were eventually calculated, with heterogeneity assessed using *I* ^2^ statistics; and values of 25%, 50% and 75% were considered low, moderate and high heterogeneity, respectively. A summary ROC (SROC) curve was plotted, incorporating individual study estimates, and confidence intervals (CIs) for sensitivity and specificity were computed using the Wilson score method. RevMan (version 5.3; Cochrane Collaboration) and Stata IC 18 software were used.

## 3 Results

### 3.1 Search Results and Selection of Included Studies

**FIGURE 1:**
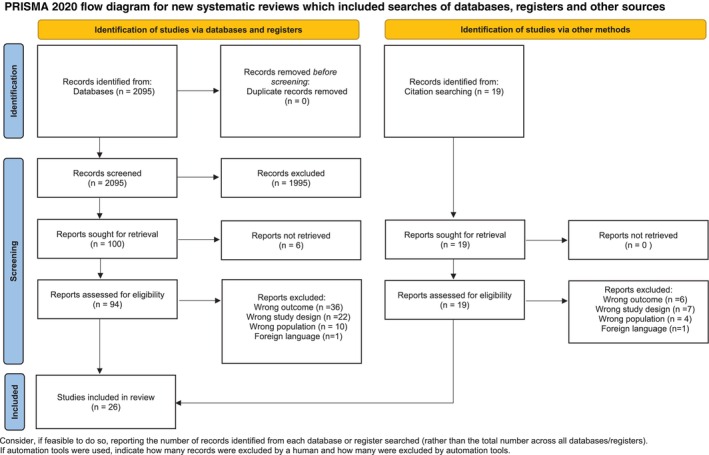
PRISMA flow diagram for PICO 1. This work is licensed under CC BY 4.0. To view a copy of this license, visit https://creativecommons.org/licenses/by/4.0/. Source: Page MJ, et al. BMJ 2021;372:n71. https://doi.org/10.1136/bmj.n71.

For PICO 1, a total of 2095 records were retrieved from database searches, with 19 additional records identified through manual search. Eventually, without retrieving any duplicates, 2114 records were screened by title and abstract; 1995 records were excluded and 113 reports were selected for further full‐text evaluation. Twenty‐six studies were included in the present work. The PRISMA flowchart illustrates the study selection process and search results (Figure 1).

**FIGURE 2:**
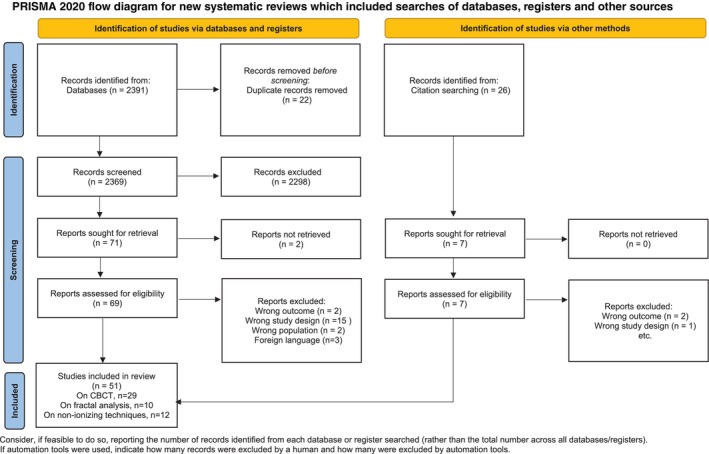
PRISMA flow diagram for PICO 2. This work is licensed under CC BY 4.0. To view a copy of this license, visit https://creativecommons.org/licenses/by/4.0/. Source: Page MJ, et al. BMJ 2021;372:n71. https://doi.org/10.1136/bmj.n71.

For PICO 2, the literature search yielded 2417 relevant records. Ultimately, 51 studies were selected (Figure 2). The kappa score calculated for screening agreement between the reviewers was 0.90 for PICO 1 and 0.92 for PICO 2.

### 3.2 Characteristics of Included Studies for PICO 1

Concerning the traditional radiography, studies investigating the accuracy of the diagnosis of periodontitis at the patient level are summarized in Table 1 and at site level in Table 2. Appendix 1 gives complete information about the included studies.

**TABLE 1:** Summary of the studies investigating the diagnostic accuracy of standard 2D radiographs at the patient level.

Author (year), country	Study design, setting	Funding	Population	Diagnosed condition and definition	Index measure	Reference measure	Test assessor	Test accuracy measure	Main conclusion
Reference measure: Clinical assessment									
Douglass et al. (1986) USA	Longitudinal cohort; University	National Center for Health Services Research and Veterans Administration Medical Research Service	Dentally asymptomatic adults from Veteran Clinic *N* = 602 Age range: 28–76 years	Bone loss to assess periodontitis (no case definition provided)	Periapical, bitewing and panoramic radiographs; Standardization: NR	Clinical examination NR	Four calibrated examiners	Sensitivity: periapical, 84.03 (0.4); panoramic, 86.4 (0.4); bitewing, 86.4 (0.4) Specificity: periapical, 80.01 (0.5); panoramic, 46.1 (0.6); bitewing, 69.1 (0.5)	Sensitivity of dental radiographs in detecting evidence of periodontal disease is relatively high. Panoramic radiographs present lower sensitivity and higher specificity in detecting periodontal disease than periapical and bitewing radiographs.
Atchison et al. (1995) USA	Cross‐sectional; University	Agency for health care policy and research	Patients seeking dental treatment *N* = 490 Age (mean ± SD): 39 ± 15 years	Bone loss to assess periodontitis based on mesial or distal BL, and FI: no precise case definition provided.	Periapical and bitewing radiographs Standardization: NR	Clinical examination NR	Three examiners	Sensitivity: 69.5% Specificity: 77%	Clinical and radiological methods should be used in combination for diagnosis.
Walsh et al. (1997) UK	Cross‐sectional; Hospital	No funding	Patients with periodontitis *N* = 50 Age (mean; range): 42; 25–61 years	Bone loss to assess periodontitis as defined based on CPITN	Panoramic radiographs Standardization: NR	Clinical examination CPITN WHO periodontal probe	Two examiners	A statistically significant relationship between CPITN and radiographic measurement.	There is a close correlation between the CPITN screening codes and bone loss on the dental panoramic radiographs.
Merchant et al. (2004) UK	Cross‐sectional; Private practice (affiliated to the Harvard school of dental medicine)	No funding	Patients with periodontitis. Number: 37 Gender (M/F): 13/24 Age (mean ± SD): 39.8 ± 12.4 years	Bone loss to assess periodontitis, defined as having at least one site with alveolar bone loss > 3, 4 and or 5 mm	Bitewing radiographs presenting the patient's dental record before the current standardized radiographs were taken	Clinical examination NR	One calibrated examiner	Sensitivity: > 3 mm (80.8, 62.1–91.5); > 4 mm (72.1, 43.4–90.3); > 5 mm (80.0, 37.6–96.4) Specificity: > 3 mm (90.9, 62.3–98.4); > 4 mm (88.5, 71.0–96.0); > 5 mm (90.6, 75.8–96.8)	The use of non‐standardized radiographs is a practical, inexpensive, reliable and valid way to assess periodontitis in epidemiological studies.
Farook et al. (2020) Saudi Arabia	Cross‐sectional; NR	NR	Patients with established periodontitis *N* = 104 Age (mean ± SD): 53 ± 10.7 years	Bone loss to assess periodontitis stages based on the 2017 WWP Classification (Tonetti et al. 2018)	Panoramic, periapical radiographs. Long‐cone paralleling technique. Standardization: NR	Clinical examination CAL measured as distance from the CEJ to the depth of the periodontal pocket or sulcus UNC‐15 periodontal probe	NR	For panoramic radiograph: A moderate positive correlation between the stages of CAL and stages of bone loss (*r* = 0.36). For periapical radiographs: stronger positive correlation (*r* = 0.50)	A combination of clinical and radiographic examinations should be performed for the accuracy of diagnosis.
Machado et al. (2020) Portugal	Cross‐sectional; Hospital	No funding	SoPHiAS participants (study of Periodontal Health in Almada‐Seixal) *N* = 456 Age (mean ± SD): 59.9 ± 15.7 years	Bone loss to assess periodontitis based on the 2017 WWP Classification (Tonetti et al. 2018) and Eke et al. (2012) case definition	Panoramic radiographs Standardization: NR	Clinical examination CAL Calibrated periodontal probe	Three calibrated and blinded examiners	Sensitivity: 99.6% (99.1–100) Specificity: 58.9 (54.4–63.4)	Radiographic BL might be a competent screening tool for periodontitis.

Abbreviations: BL, bone loss; CAL, clinical attachment level; CPITN, community periodontal index treatment need; FI, furcation involvement; NR, not reported; WHO, World Health Organization.

**TABLE 2:** Summary of the studies investigating the diagnostic accuracy of standard 2D radiographs at the site level.

Author (year), country	Study design, setting	Funding	Population	Diagnosed condition and definition	Index measure	Reference measure	Test assessor	Test accuracy measure	Main conclusion
Studies focusing on intrabony defects									
Reference measure: Intra‐surgical assessment									
Eickholz et al. (1999) Germany	Cross‐sectional study; University	NR	Patients with moderate to advanced untreated periodontal disease *N* = 33 Age range: 28–68 years	Intrabony defects (*n* = 50)	Standardized bitewing radiographs performed with modified film holders Digital filtering Tube voltage: 70 kV Tube current: 7 mA	Intra‐surgical measurements Distances from CEJ to AC and CEJ to BD (deepest extension of the bony defect) PCPUNC 15 periodontal probe	Single blinded calibrated examiner	Mean differences between radiographic and intra‐surgical measurements: – CEJ to AC: ±0.12 mm (overestimation/underestimation) – CEJ to BD: Underestimation of 0.3–0.8 mm	Digital radiographs, whether unaltered or with basic manipulations, provide measurements close to the intra‐surgical gold standard but consistently underestimate interproximal bone loss. Specific filters may reduce validity.
Zybutz et al. (2000), USA	Longitudinal study; USA	Supported by NIH grant P30 DEO9743 and a grant from Guidor USA	Patients with interproximal intrabony defects *N* = 29 NR	Intrabony defects (*n* = 57)	Standardized intraoral periapical radiographs	Intra‐surgical measurements Alveolar bone level (ABL): Distance from CEJ to bottom of the intra‐bony defect. Intra‐bony defect depth (IBD): Distance from interproximal bone crest to the bottom of the defect CP‐15 UNC periodontal probe	Two blinded calibrated examiners	Radiographs underestimated defect depth by ~1.4 mm compared to surgical measurements. Probing to bone underestimated/overestimated surgical measures within 0.5 mm in 39% of cases. – ICC values: Probing to bone vs. surgical: 0.87 Radiographs vs. surgical: 0.61	Radiographs underestimated bone levels and defect depths but are reliable for assessing changes over time. Probing to bone provided accurate baseline measures and can substitute for re‐entry procedures.
Pepelassi et al. (2000), Greece	Cross‐sectional study; Departments of Periodontology and Oral Diagnosis, University of Athens	NR	Patients diagnosed with generalized chronic periodontitis, as defined by the 1999 Classification *N* = 100 Age range: 18–75 years	Intrabony defects (*n* = 1049)	Periapical and panoramic radiographs Standardization: NR	Intra‐surgical measurements Depth: Alveolar crest to defect base. Mesiodistal width: Mesial to distal borders at crest. Buccolingual width: Buccal to lingual/palatal borders at crest UNC‐15 peridontal probe	Calibrated examiners	Findings: – Periapical radiography detected 61.85% of defects; panoramic radiography detected 20.99%. – Defect detection depended on defect depth, bucco‐lingual width, number of walls, and jaw location. – Periapical radiography was more accurate in assessing defect depth and mesio‐distal width compared to panoramic radiography	Periapical radiography is superior to panoramic radiography in detecting and measuring periodontal endosseous defects. Panoramic radiography is not recommended for precise evaluation.
Wolf et al. (2001), Germany	Cross‐sectional study; University	Support by Friadent Company for software/hardware	Patients with moderate to advanced untreated periodontal disease *N* = 50 Age range: 22–65 years	Intra‐bony defects (*n* = 50)	Standardized bitewing radiographs performed with modified film holders Digital filtering and magnification Tube voltage: 70 kV Tube current: 7 mA	Intra‐surgical measurements Distance from the CEJ to the alveolar crest (AC). Distance from the CEJ to the deepest extent of the bony defect (BD) PCPUNC 15 periodontal probe	Two independent and blinded examiners	Mean differences: – CEJ‐AC: Overestimation by 0.74–1.91 mm – CEJ‐BD: Overestimation by 0.04–0.77 mm Filters failed to improve reproducibility or validity significantly	Un‐manipulated digital radiographs closely approximated intra‐surgical measurements. Manipulations did not improve measurement accuracy and sometimes reduced validity.
Hörr et al. (2005) Germany	Cross‐sectional study; University	NR	Patients with moderate to advanced untreated periodontitis *N* = 34 Age range: 22–65 years	Infra‐bony defects (*n* = 50)	Standardized bitewing radiographs performed with modified film holders Digital filtering Tube voltage: 70 kV Tube current: 7 mA	Intra‐surgical measurements Distances from CEJ to AC, CEJ to BD (deepest extension of the bony defect), height of the infrabony component (INFRA) PCPUNC 15 periodontal probe	Single trained examiner	CEJ‐AC: Small overestimation (0.35–0.68 mm), influenced by vertical angulation difference (*p* = 0.047) and intra‐surgical CEJ‐AC (*p* = 0.003). CEJ‐BD: Underestimation (0.84–1.12 mm), influenced by intra‐surgically assessed bone loss (*p* = 0.012). INFRA: Underestimation (0.66–0.76 mm), influenced by individual patient, intra‐surgically assessed INFRA (*p* < 0.001), and digital filter applied (*p* = 0.018)	Digital manipulations do not increase accuracy of alveolar bone loss assessment. Overall measurements on digitised un‐manipulated and filtered images are quite close to intra‐surgical measurements.
Pahwa et al. (2014) India	Cross‐sectional study; University	NR	Patients with generalized moderate to severe chronic periodontitis *N* = 15 Age range: 20–54 years Mean age: 38 years	Intrabony defects	Radiovisiograph (RVG) Long‐cone paralleling technique Tube voltage: 70 kVp Tube current: 8 mA Standardization: NR	Intra‐surgical measurements Measured as CEJ to base of defect (BD) and CEJ to alveolar crest (AC) UNC‐15 periodontal probe	Single blinded examiner	Mean differences: RVG: 0.645 mm (CEJ‐BD), underestimation in 96.8% of sites ICC: – RVG: 0.851 (95% CI: 0.194–0.954)	CT scans provided the closest agreement with surgical measurements, outperforming RVG for accuracy in intrabony defect assessment.
Saberi et al. (2017) Iran	Cross‐sectional study; University	No funding	Patients with chronic periodontitis requiring surgery *N* = 60 Mean age: 44.5 years	Intrabony defects (*n* = 90)	Digital panoramic radiographs Exposing factors adjusted according to age and bulk to prevent magnification effect Standardisation: NR	Intra‐surgical measurements Distance CEJ‐alveolar bone margin Williams probe, acrylic stent to standardize probing angulation	Oral radiologist (for radiographic measurements), periodontist (for surgical measurements)	High correlation between bone probing and surgery (*r* = 0.98). Moderate to high correlation between digital ruler (*r* = 0.89) and digital calliper (*r* = 0.79) with surgery. Both radiographic methods significantly underestimated defect depth compared to surgery	Information obtained from digital panoramic radiographs should be used with caution. Measuring the defects on panoramic radiographs underestimated the depths.
Studies focusing on furcation involvement (FI)									
Reference measure: clinical assessment									
Alasqah et al. (2022) Saudi Arabia	Cross‐sectional study using retrospective data University	No funding	Patients seeking for periodontal treatment *N* = 52 Mean age: 39.6 (SD: 10.1) years	Furcation involvement classified according to the Hamp classification	Radiographs Standardization: NR	Clinical examination Measurement of horizontal loss of periodontal support tissue (Hamp's classification) Nabers probe	Single calibrated examiner	Sensitivity: 72.8% Specificity: 70.7%	Radiographic assessment underestimates furcation involvement compared to clinical evaluation, showing slightly better reliability for Classes II and III furcations. Radiographic visibility is more apparent when clinical PD and CAL exceed 5 mm.
Reference measure: intra‐surgical assessment									
Deas et al. (2006) USA	Cross‐sectional study; Wilford Hall Medical Center (WHMC), Lackland Air Force Base	NR	Patients with moderate to advance periodontitis *N* = 89 Mean age: NR	FI classified according to the Hamp classification (*n* = 328)	Periapical and bitewing radiographs Standardization: NR	Intra‐surgical measurements Measurement of horizontal loss of periodontal support tissue (Hamp's classification) Nabers 2N probe	Five independent examiners	Sensitivity: 38.7%; Specificity: 92.2%. PPV: 71.7% NPV: 74.6%	‘Furcation Arrow’ image ‘per se’ has limited usefulness as a diagnostic marker and is often difficult to interpret There is high variability among clinicians about what constitutes a furcation arrow image.
Yusof et al. (2021) Malaysia	Parallel, single‐blinded, randomized controlled trial (RCT) University	LESTARI Research Grant, Universiti Teknologi MARA, Malaysia	Patients with Stage III/IV periodontitis^a^ with FI Class II or III requiring surgery. *N* = 22 Mean age range: 44–47.8 years	FI in the horizontal and vertical components	Periapical radiographs Long‐cone paralleling technique Standardization: NR	Intra‐surgical measurements Vertical and horizontal bone loss (BL‐V; BL‐H), furcation width (FW), root trunk length (RT) and clinical attachment level (CAL) PCPUNC‐15 periodontal probe	Trained calibrated examiners	The Z‐statistics comparing radiographic and surgical measurements for various parameters revealed no significant differences, with the exception of vertical bone loss in the maxillary molars	Periapical radiograph are less accurate for vertical bone loss measurement of maxillary molar with furcation defects.
Studies focusing on marginal bone crest									
Reference measure: clinical assessment									
Papapanou and Wennström (1989) Sweden	Cross‐sectional study; Department of Periodontology, University of Gothenburg	Supported by the Swedish Medical Research Council and Colgate‐Palmolive	Patients with periodontitis *N* = 191 subjects, Age range: 35–80 years	Marginal bone loss (*n* = 4682 approximal tooth sites)	Periapical and vertical bitewing radiographs Standardization: NR	Clinical examination Distance from the CEJ to the most apical extent of clinical attachment loss recorded to the nearest millimetre Calibrated periodontal probe	Measurements performed by trained examiners; radiographs interpreted by experienced radiologists	Strong correlation between radiographic and clinical measurements (*r* = 0.80, *p* = 0.0001). 92% of sites showed differences ≤ 2 mm between methods. Agreement worsened in sites with severe periodontal breakdown (bone loss > 12 mm).	Both radiographic and clinical methods provide reliable assessments of periodontal destruction, but discrepancies increase in severe cases. Radiographs slightly overestimate bone loss compared to probing.
Rams et al. (1994) USA	Longitudinal before/after study University	NIDR Grant	Patients previously treated for periodontitis on maintenance care over a 36‐month period. *N* = 51 Mean age: NR	Marginal bone loss (*n* = 2332 interproximal tooth sites)	Periapical and bitewing radiographs Long‐cone paralleling technique Standardization: NR	Clinical examination PAL, measured relative to the occlusal stent, interproximal sites. Measurements considered both: A ≥ 2 mm loss in PAL combined with a ≥ 2 mm increase in probing depth. A ≥ 3 mm increase in probing depth alone	A single periodontist	Sensitivity: presence, 16.9%–17.0%; absence, 100%–87.3% Specificity: presence, 100%–87.3%; absence, 16.9%–16.9%	The diagnostic value of conventional dental radiographs can be expanded with evaluation of crestal lamina dura status at interproximal tooth sites, as the evaluation of radiographic crestal lamine dura status appears valuable for assessing the risk of periodontitis disease‐activity.
Hausmann et al. (1994) USA	Longitudinal before/after study University	NIH Grant	Patients with established periodontitis *N* = 52 Age range: 28–62 years	Marginal bone loss (*n* = 1972)	Periapical and bitewing radiographs Long‐cone paralleling technique Standardization: NR	Clinical examination PAL; thresholds for significant loss: molars ≥ 2.18 mm, non‐molars ≥ 1.90 mm Florida Probe	NR	Correlation between radiographic and probing changes (*r* = 0.17).	Weak correlation between change in probing attachment and change in radiographic bone height on an individual site.
Khocht et al. (1996) USA	Cross‐sectional study; University	NR	Patients seeking for diagnosis *N* = 23 Age range: 18–65 years	Marginal bone loss	Periapical and bitewing radiographs Long‐cone paralleling technique (for periapical) Standardization: NR	Clinical examination PPD, six sites per tooth measurements were recorded to the nearest millimetre; CAL, calculated as the sum of PPD and the position of the gingival margin relative to the CEJ Williams probe	Two independent examiners	Correlation between probing depths and PSR scores (*r* = 0.704). Correlation between attachment levels and PSR scores (*r* = 0.62). Correlation between attachment levels and BW films (*r* = 0.554). Correlation between attachment levels and PA films (*r* = 0.328)	Radiographs taken in clinical practice situations are not highly reflective of the periodontal status as determined by probing depth and attachment level.
Machtei et al. (1997) USA	Longitudinal before/after study; Private practice	USPHS Grant	Patients with untreated moderate/advanced periodontitis *N* = 79 Mean age: 45 (SD: 9.1) years	Angular or horizontal marginal bone morphology (*n* = 2950)	Periapical and bitewing radiographs Long‐cone paralleling technique Standardization: NR	Clinical examination CAL measured as the distance from the CEJ to the base of the clinical pocket Florida probe	NR	Sensitivity: 16% (Rx, site level); 51% (Rx, patient level) Specificity: 81% (Rx, site level); 55% (Rx, patient level)	CAL and radiographic bone height change independently in the short term but converge over time. Both methods are useful for monitoring disease and treatment.
Kim et al. (2008) Germany	Cross‐sectional study; University	NR	Patients with severe chronic periodontitis or aggressive periodontitis *N* = 110 Age range: 25–64 years	Marginal bone loss	Periapical and panoramic radiographs Standardization: NR	Clinical examination NR	Two calibrated examiners	Agreement between intraoral and panoramic radiographs was observed in 50%–80% of teeth. Differences greater than 10% of the total root length were noted in 20%–50% of teeth. Significant differences were particularly observed in maxillary molars, premolars and mandibular central incisors	Panoramic radiographs can either overestimate or underestimate linear distances, with significant differences between imaging techniques. Replacing intraoral periapical radiographs with panoramic radiographs is not recommended for patients with severe chronic or aggressive periodontitis.
Gedik et al. (2008) Turkey	Longitudinal cohort study NR	NR	Patients planned for surgical subgingival curettage *N* = 21 Mean age (range): 35.7 (18–59) years	Crestal alveolar bone level (*n* = 42)	Periapical, bitewing and panoramic radiographs Intraoral radiographs: Tube voltage: 70 kVp Tube current: 7 mA Exposure time: 3.5 s Standardization: NR	Clinical examination PPD Williams probe	One examiner	NR	Bitewing radiography is superior to periapical, with only a slight difference compared to panoramic. Panoramic and bitewing would be preferable to periapical images for crestal bone assessment.
Ashwinirani et al. (2015) India	Cross‐sectional study; NR	No funding	Patients with moderate to severe chronic periodontitis requiring periodontal surgery *N* = 30 Mean age: 43 (SD: 7.9) years	Interdental alveolar bone loss	Periapical radiographs (IOPA) and radiovisiography (RVG) Long‐cone paralleling technique IOPA: Film size: No. 2 Tube voltage: 65 kVp Tube current: 7.5 mA Exposure time: 0.5 s RVG: Tube voltage: 65 kVp Tube current: 7.5 mA Exposure time: 0.06 s Standardization: occlusal stent	Intra‐surgical measurements Bone loss measured from reference points (occlusal stent) to the crest (MC, DC) and base (MB, DB) of the bone on mesial and distal sides UNC 15 periodontal probe	Single oral and maxillofacial radiologist	Significant differences were found between periapical radiographs and intra‐surgical measurement, and between RVG and intra‐surgical measurements. No difference between the two radiographic techniques	IOPA and RVG are useful in detecting interdental bone loss but underestimated it by 1.5–2.5 mm. RVG is superior to IOPA due to reduced time and radiation exposure.
Rams et al. (2018) USA	Retrospective longitudinal cohort study; University	Partially funded by grants from the National Institute of Dental and Craniofacial Research, and the Paul H. Keyes Term Professorship in Periodontology	Treated patients on periodontal maintenance care *N* = 56 Mean age: 54.2 (SD: 11.8) years	Angular and horizontal marginal bone morphology(*n* = 1356 interproximal sites)	Periapical and bitewing radiographs Long‐cone paralleling technique Standardization: NR	Clinical examination Assessment of progressive periodontitis defined by a PPD increase of ≥ 3 mm, or *a* ≥ 2 mm increase in PPD with ≥ 2 mm loss in CAL, measured from an occlusal reference stent	Independent examiners	Intra‐examiner reproducibility: Exact agreement: 97.9% Cohen's kappa (*κ*): 0.80 ± 0.06 Inter‐examiner reproducibility: Exact agreement: 97.0% Cohen's kappa (*κ*): 0.76 ± 0.07	There is a moderate correlation between clinical attachment level and radiographically assessed bone level, but with variations depending on the tooth type.
Reference measure: Intra‐surgical assessment									
Åkesson et al. (1992) Sweden	Cross‐sectional study; University	Supported by the Swedish National Institute of Radiation Protection	Patients with advanced periodontitis *N* = 23 Age range: 27–73 years Mean age: 49 years	Marginal alveolar bone levels	Panoramic, bitewing and periapical radiographs Standardization: occlusal splint	Intrasurgical measurements Distance between the metal thread on a custom splint (reference point) and the bone level was recorded to the nearest millimetre Extended periodontal probe (34 mm)	Five experienced examiners	All radiographic methods underestimated bone loss. Periapical radiography was most accurate, deviating 13% from the true value. Panoramic radiography had the lowest accuracy with 13%–32% underestimation. Probing before surgery was most accurate, deviating only ~5% from surgical measures.	Periapical radiography is superior to panoramic and bitewing for measuring marginal bone levels. Clinical probing is most accurate but invasive.

Abbreviations: AC, alveolar crest; CAL, clinical attachment level; CEJ, cemento‐enamel junction; FI, furcation involvement; IOPA, intraoral periapical radiograph; NPV, negative predictive value; NR, not reported; PAL, probing attachment level; PPD, probing pocket depth; PPV, positive predictive value; RVG, radiovisiography.

### 3.3 Characteristics of Included Studies for PICO 2

Concerning the alternative and emerging imaging techniques, studies investigating the accuracy of CBCT are summarized in Table 3, those exploring the role of fractal analysis in Table 4, those exploring digital methods applied to analyse radiographic (ionizing) imaging in Table 5 and those exploring the diagnostic performance and application of non‐ionizing imaging methods in Table 6. Appendix 1 gives complete information about the included studies.

**TABLE 3:** Summary of the studies investigating the diagnostic accuracy of CBCT.

Author (year), country	Study design, setting	Funding	Population	Diagnosed condition and definition	Index test	Reference test	Test assessor	Test accuracy measure	Main conclusion
Studies focusing on intra‐bony defects									
Grimard et al. (2009) USA	Cross‐sectional nested in an RCT; University	NR	Patients with periodontitis *N* = 29 Age range: 35–65	Intrabony defects (*n* = 35) with probing depth ≥ 5 mm and vertical defect ≥ 3 mm	CBVT FOV: NR Voxel size: NR Tube voltage: NR Tube current: NR Scanning time: 18 s Slice thickness: 1 mm	Digital intraoral radiographs CEJ‐AC CEJ‐BD Intra‐surgical measurements CEJ‐AC CEJ‐BD 15 mm periodontal probe	Two board‐certified periodontitis for the clinical measurement. One examined for radiographic measurements.	CBVT strongly correlated with surgical measurements (*r* = 0.89–0.95), whereas intraoral radiograph correlated less favourably (*r* = 0.53–0.67)	Compared to direct surgical measurements, CBVT was significantly more precise and accurate than intraoral radiographs.
de Faria Vasconcelos et al. (2012) Brazil	Cross‐sectional; Private dental radiology clinic	NR	Patients undergoing imaging for periodontal assessment *N* = 11 Age range: 39–66 years	Combined bone defects (*n* = 39), identified according to the classification of Goldman and Cohen	CBCT FOV: 6 cm Voxel size: 0.2 mm Tube voltage: 120 kV Tube current: 3–6 mA Scanning time: NR Slice thickness: NR	Periapical radiographs CEJ‐AC, CEJ‐BD, AC‐dental root adjacent to the defect (width of the defect) Image tool software ruler	Three trained examiners: one radiologist and two Master's students	No statistically significant differences in identification of the pattern of alveolar bone loss in either imaging modality. However, they differ as to the measurement of the height of the alveolar bone crest	CBCT allowed the identification of combined bone defects through a 3D evaluation of the alveolar bone crest.
Raichur et al. (2012) India	Cross‐sectional; Dental school	NR	Patients with moderate to severe chronic periodontitis scheduled for periodontal surgery *N* = 28 Mean age: 45 (range: 30–60) years	Infrabony defects (*n* = 28); definitions based on CEJ‐AC and CEJ‐BD measurements	RVG (radiovisiography) and DVT (digital volume tomography) FOV: 50 × 37 mm Voxel size: NR Tube voltage: 70–74 kV Tube current: 10 mA Scanning time: 10.8 s Slice thickness: NR	Intra‐surgical measurements CEJ‐AC, CEJ‐BD from buccal/labial and lingual palatal aspects of each defect UNC 15 probe	NR	No differences in the mean measurements between RVG, DVT, SUR techniques in the CEJ‐AC group whereas the same measurements showed a significant difference in the CEJ‐BD group	DVT technique was significantly more accurate than RVG in detecting intrabony periodontal defects.
Moradi Haghgoo et al. (2014) Iran	Cross‐sectional; Dental school	Dentistry Faculty, Hamadan University of Medical Sciences	Patients seeking periodontal treatment NR Mean age: NR	Intrabony defect (*n* = 50) Sites with interproximal osseous defects (horizontal or 3 wall vertical)	CBCT FOV: 6 cm Voxel size: 200 μm. Tube voltage: NR Tube current: 10.6 mA Scanning time: NR Slice thickness: 1 mm	Digital intraoral radiographs CEJ‐depth of the defect Image tool software ruler Intra‐surgical measurements CEJ‐defect depth Williams probe and rubber stop	Two examiners who evaluated the images at 2‐week interval. No information on intra‐surgical measurements	Discrepancy from the mean of the intra‐surgical measurements (gold standard) was lower in CBCT. Smaller deviation range for the CBCT measurements compared to the intraoral radiographs in the anterior and posterior regions. Better accuracy of CBCT in evaluating vertical defects than intraoral radiographs	CBCT and digital radiographs can be used in periodontal bone assessments. CBCT showed better accuracy.
Banodkar et al. (2015) India	Cross‐sectional; University	NR	Patients with periodontitis with bony defect requiring surgery *N* = 15 Mean age: NR	Periodontal bony defects (*n* = 100; 80 horizontal defects; 20 vertical defects)	CBCT FOV: 4 × 4 cm Voxel size: 400 μm Tube voltage: 90 kV Tube current: 10 mA Scanning time: 13 s Slice thickness: NR	Intra‐surgical measurements CEJ‐AC (horizontal defects) CEJ‐BD (vertical defects) Endodontic reamer	NR	Very high correlation (0.988) between the surgical and CBCT measurements. The correlation was higher in horizontal defects as compared to vertical defects although the difference was not significant	CBCT is highly accurate in identifying and quantifying bone loss for both horizontal and vertical defects.
Li et al. (2015) China	Cross‐sectional; University	Beijing Municpal Science and Technology Plan the Capital Characteristic Clinical Application Research	Patients with generalized aggressive or advanced chronic periodontitis requiring periodontal surgery. *N* = 44 Mean age: 39 years	Intrabony defects (*n* = 44)	CBCT FOV: NR Voxel size: NR Tube voltage: 110 kV Tube current: 12–17 mA Scanning time: NR Slice thickness: 0.5 mm	Periapical radiographs CEJ‐BD BD‐AC (depth) M‐D (width of the intrabony defect) Intra‐surgical measures CEJ‐BD BD‐AC (depth) M‐D (width of the intra‐bony defect)	Clinical measurements by experienced periodontists. Radiographic measurement by one calibrated examiner.	ICC for reproducibility of CBCT measurements: 98%. CBCT measurements of the width of the defect were similar to the intra‐surgical measurements. Conversely, the depth of the defect was generally underestimated by CBCT	CBCT provides relatively accurate measurements of the width of the intra‐bony defect. For vertical measurements, CBCT seems to provide no advantages over conventional periapical radiographs.
Guo et al. (2016) China	Cross‐sectional, University	Beijing Municipal Science & Technology Commission	Patients seeking periodontal treatment requiring surgery *N* = 6	Intrabony defect (*n* = 150) on premolars and molars (*n* = 25) assessed on alveolar bone level (distance between the CEJ and the apical base of the periodontal bone defect)	CBCT FOV: 4 × 4 cm Voxel size: 0.125 mm Tube voltage: 75–85 kV Tube current: 5 mA Scanning time: NR Slice thickness: 1 mm	Intra‐surgical measurements CEJ‐ ABD (apical base of the periodontal bone defect) UNC 15 probe	Four trained observers, for CBCT measurements. One experienced periodontitis for intra‐surgical measurements	Diagnostic coincidence rates of four observers were 86.7%, 87.3%, 88.7% and 88.0%. There was no significant difference between CBCT and intra‐surgical measurement	The measuring method in CBCT images provides useful and accurate information for evaluating alveolar bone levels, with high diagnostic coincidence rates and minimal observer variability
Suphanantachat et al. (2017) Thailand	Cross‐sectional; University	Faculty Research Fund, Faculty of Dentistry, Chulalongkorn University, Bangkok, Thailand	Patients with moderate to severe periodontitis and at least two intrabony defects. *N* = 25 Mean age: 48.8 (SD: 9.3) years	Intrabony defects (*n* = 116/666 teeth) defined as a defect of ≥ 3 mm depth on a periapical radiograph	CBCT FOV: 100 × 100 mm Voxel size: 0.25 mm Tube voltage: 80 kV Tube current: 5 mA Scanning time: 17.5 s Slice thickness: NR	Clinical examination CAL Periapical radiographs NR	Single operator for clinical measurements. Three calibrated examiners for CBCT measurements.	The agreement between CBCT and periapical radiograph for periodontal diagnosis was 79.3%. The concordance on the infrabony defect classification was 44.7%	CBCT offers additional benefits over the traditional periapical radiographs on periodontal and infrabony defect assessment.
Adurty et al. (2021) India	Cross‐sectional University	NR	Patients with chronic and aggressive periodontitis with intrabony defects *N* = 25 Mean age: 35 years	Intra‐bony defects	CBCT FOV: 5 × 5 cm Voxel size: 0.9 mm Tube voltage: 80 kV Tube current: 10 mA Scanning time: 12 s Slice thickness: NR	Clinical examination CAL Digital periapical radiographs CEJ‐AC CEJ‐BD AC‐dental root adjacent to the defect (width of the defect) Defect angle	Postgraduates under supervision by senior periodontists made the clinical measurements. Two examiners made the radiographic measurements	In detection of the bone loss patterns, no statistical difference was seen between the two techniques	Intrabony morphological parameters were similarly measured on digital periapical radiographs and CBCT.
Nikolic‐Jakoba et al. (2021) Serbia	Cross‐sectional; University	Ministry of Education, Science, and Technological Development of the Republic of Serbia	Patients with generalized severe periodontitis with at least 2 intrabony defects requiring periodontal surgery. *N* = 21 Mean age: 44.8 (SD: 8.37) years	Intrabony defect on premolars and molars (*n* = 66)	CBCT FOV: 80 × 100 mm Voxel size: 0.25 mm Tube voltage: 90 kV Tube current: 10 mA Scanning time: 2.4 s Slice thickness: 0.25, 1.00 and 3.00 mm	Clinical examination CAL Intra‐surgical measurements Clinical bone level (cBL) measured as distance between tip of K file and reference stent K file #60	A single trained and calibrated observer for the clinical measurements. Two calibrated observers for the CBCT measurements	No significant difference between the clinical and the radiographic measurements, independent of CBCT section thickness	CBCT measurements can be considered clinically valid and reliable.
Studies focusing on furcation involvement (FI)									
Walter et al. (2009) Switzerland	Cross‐sectional; University	No funding	Patients with generalized chronic periodontitis *N* = 12 Mean age: age of 57.5 (range: 41–80) years	FI in maxillary molars (*n* = 22) according to Hamp et al. classification (Hamp et al. 1975)	CBCT FOV: 4 × 4–6 × 6 cm Voxel size: NR Tube voltage: 74–90 kV Tube current: 5–8 mA Scanning time: NR Slice thickness: NR	Clinical examination Measured at three sites (buccal, mesio‐ palatal and disto‐palatal) according to Hamp's classification PQ2N Nabers probe Periapical radiographs	Two trained and calibrated periodontists	FI was clinically observed in all 66 furcation entrances, while a FI degree I–III was found in 52 sites according to the CBCT. Only 27% of the clinical findings were confirmed by CBCT	CBCT add substantial information about the root form and proximity, and FI at maxillary molar teeth.
Walter et al. (2010) Switzerland	Cross‐sectional; University	No funding	Patients with generalized chronic periodontitis *N* = 14 Mean age: 57.0 (range: 42–81) years	FI in maxillary molars (*n* = 25) according to Hamp et al. classification (Hamp et al. 1975)	CBCT FOV: 4 × 4–6 × 6 cm Voxel size: 0.08–0.25 mm Tube voltage: 74–90 kV Tube current: 5–8 mA Scanning time: NR Slice thickness: NR	Intra‐surgical measurements Measured at three sites (buccal, mesio‐ palatal and disto‐palatal) according to Hamp's classification PQ2N Nabers probe	Two trained and calibrated periodontists	CBCT revealed an FI in 59 out of 75 furcation entrances, and 84% of the CBCT data were confirmed by the intra‐surgical findings (Kappa ranging from 0.89 to 0.95).	CBCT of the FI of maxillary molars have a high degree of agreement with those from intra‐surgical assessments.
Marinescu et al. (2014) Romania	Cross‐sectional, NR	NR	Patients with generalized advanced chronic periodontitis *N* = 19 Mean age: 45.48 (range: 23–65) years	FI in lower molars (*n* = 25), classified according to Hamp et al.'s	CBCT FOV: 12 × 15 × 15 cm Voxel size: NR Tube voltage: 85 kV Tube current: 5–7 mA Scanning time: NR Slice thickness: NR	Clinical examination Hamp's classification Nabers probe	One experienced examiner for clinical measurements and one experienced operator for CBCT analysis	Clinical measurements were confirmed in 84% of cases by CBCT. CBCT accurately detected Grades II and III FI but early defects were more challenging to diagnose	CBCT is effective in detecting advanced furcation defects and provides detailed information about defect dimensions and anatomy. However, it does not yet replace clinical measurements.
Qiao et al. (2014) China	Cross‐sectional; University	No external funding; supported by the authors' institution	Patients with generalized chronic periodontitis *N* = 15 Mean age: 43.5 years	FI in maxillary molars (*n* = 51) defined according to Hamp et al. (1975)	CBCT FOV: 4 × 4–6 × 6 cm Voxel size: 0.125 mm Tube voltage: 74–90 kV Tube current: 5–8 mA Scanning time: NR Slice thickness: 0.5 mm	Intra‐surgical assessment Measured at three sites (buccal, mesio‐ palatal and distopalatal) according to Hamp's classification PQ2N Nabers probe	Two trained and calibrated periodontists for clinical measurements; two trained radiographers for CBCT evaluation	CBCT data compared with intra‐surgical findings; overall agreement was 82.4% with a weighted kappa of 0.917	CBCT provides a highly accurate assessment of maxillary molar furcation involvement, offering detailed information on root morphology and lesion severity, which surpasses conventional clinical assessments.
Cimbaljevic et al. (2015) Serbia	Cross‐sectional University	NR	Patients with generalized chronic periodontitis with at least 2 intrabony defect. *N* = 15 Mean age: 44.5 years	FI on maxillary (*n* = 20) and mandibular (*n* = 36) molars	CBCT FOV: 80 × 100 mm Voxel size: 0.25 mm Tube voltage: 90 kV Tube current: 10 mA Scanning time: 2.4 s Slice thickness: NR	Clinical examination Measured at three sites (upper molars, buccal, mesio‐ palatal and disto‐palatal) or two sites (lower molars, buccal and oral) PQ2N Nabers probe	Two independent, calibrated, experienced periodontists for clinical measurement, and two examiner for CBCT.	Good inter‐observer agreement for FI detection on CBCT (72.6%). The agreement between the clinical and CBCT evaluation of FI ranged between 43% and 55%	Clinical experience and CBCT proficiency do not have an impact on FI detection on CBCT images.
Pajnigara et al. (2016) India	Prospective cohort; Dental college	NR	Patients with generalized chronic periodontitis with at least one molar with a Grade II FI *N* = 40 Mean age: 38.05 (SD: 4.77) years	FI Grade II (*n* = 200) – horizontal and vertical components	CBCT NR	Clinical examination Measurements of vertical (CEJ as reference) and horizontal component Williams probe, Nabers probe Intra‐surgical measurement Measurements of vertical (CEJ as reference and occlusal stent) and horizontal component Williams probe, Nabers probe	Single calibrated examiner for the clinical measurement and another calibrated examiner for the CBCT measurement	CBCT measurements underestimate intra‐surgical measurements but similar to clinical measurements	CBCT appears accurate for the diagnosis of FI
Zhu and Ouyang (2016) China	Cross‐sectional University	NR	Patients seeking periodontal treatment and having FI Class II *N* = 11 Mean age: NR	FI on maxillary molar (=21) presenting with class II FI (*n* = 39) according to the Glickman classification	CBCT FOV: 4 × 4 to 6 × 6 cm Voxel size: 0.125 mm Tube voltage: 80 kV Tube current: 5–6.3 mA Scanning time: NR Slice thickness: 1 mm	Clinical examination NR	One examiner for CBCT	High reproducibility on all measured parameters in CBCT images. (ICC range: 0.960–0.992)	CBCT images provide more details in assessing maxillary molar FI compared to probing.
Padmanabhan et al. (2017) India	Cross‐sectional University	NR	Patients with moderate or severe chronic periodontitis with at least one with Grade II or III FI requiring periodontal surgery *N* = 14 Age range: 20–60 years	FI on mandibular molars (*n* = 25) according to Glickman's classification	CBCT FOV: NR Voxel size: 180 μm Tube voltage: 84 kV Tube current: 5 mA Scanning time: 20 s Slice thickness: NR	Intra‐surgical measurements Height: Furcation fornix‐alveolar base. Width: Maximum root separation above AC **Depth:** AC‐interradicular bony resistance Calibrated digital vernier calliper	Single calibrated examiner for clinical measurements. For CBCT, wo calibrated examiners	The mean values of the two methods are statistically similar	Accuracy of assessment of mandibular molar FI by CBCT was comparable to that of direct surgical measurements.
Zhang et al. (2018) USA	Cross‐sectional; University	Research Office, University of Texas School of Dentistry at Houston	Patients with generalized moderate or severe periodontitis *N* = 83 Mean age: 59.03 (SD: 13.08)	FI on first maxillary and mandibular molars according to modified Glickman's classification	CBCT FOV: 150 × 90 mm Voxel size: 0.2 mm Tube voltage: 90 kV Tube current: 10 mA Scanning time: 16 s Slice thickness: 1 mm	Clinical examination Modified Glickman's classification Periapical or bitewing radiographs Presence of triangular radio lucency at the furcation area recorded dichotomously (yes/no)	Pre‐doctoral dental students under supervision	When CBCT demonstrated no FI, 18.7% of these cases were documented as FI on clinical detection	Compared with periapical radiographs, CBCT appeared to have higher correlation coefficients with clinical detection, especially at distal palatal side of maxillary first molar.
Komšic et al. (2019) Croatia	Cross‐sectional; University	NR	Patients with generalized Stages II–IV, Grade B or C periodontitis (Tonetti et al. 2018) scheduled for periodontal surgery. *N* = 6 Mean age: 53.5 (SD: 14.8) years	FI on maxillary and mandibular molars (*n* = 38) classified according to Glickman's classification	CBCT FOV: 1001 × 999 mm Voxel size: 0.2 mm Tube voltage: 90 kV Tube current: 10 mA Scanning time: 18 s Slice thickness: 1 mm	Clinical examination Glickman's classification Nabers probe Panoramic radiograph Presence of triangular radio lucency at the furcation area recorded dichotomously (yes/no) Intra‐surgical measurement Measured at three sites or two sites, according to modified Glick‐ man's classification Nabers probe	A single investigator	CBCT showed high agreement with intra‐surgical measures: Sensitivity: 93% Specificity: 100% PPV: 100% NPV: 93% AUC: 96%	CBCT exhibits excellent agreement and higher accuracy and therefore it is clinically relevant in the FI assessment.
Yusof et al. (2021) Malaysia	RCT; University	LESTARI Research Grant, Universiti Teknologi MARA, Malaysia	Patients with Stage III/IV periodontitis (Tonetti et al. 2018) with FI Class II or III requiring surgery. *N* = 22 Mean age range: 44–47.8 years	FI in the horizontal and vertical components	CBCT FOV: 5 × 3 cm Voxel size: 0.076 mm Tube voltage: 78 kV Tube current: 10 mA Scanning time: 10.8 s Slice thickness: 0.076 mm	Intra‐surgical measurements Vertical and horizontal bone loss (BL‐V; BL‐H), furcation width (FW), root trunk length (RT), and clinical attachment level (CAL) PCPUNC‐15 periodontal probe Periapical radiograph BL‐V; BL‐H FW RT	Trained calibrated examiners	High degree of agreement between CBCT measurements and intra‐surgical findings AUC: 62.8%	CBCT provided better sensitivity of the furcation defects that periapical radiograph. CBCT‐based data were able to accurately reflect the real state of furcation defects in molar with FI.
Alsakr et al. (2022) Saudi Arabia	Cross‐sectional Dental school	NR	Patients with furcation defects in maxillary molars. *N* = 40 Mean age: 56.6 years	FI on maxillary molars—vertical component according to Tarnow classification	CBCT NR	Clinical examination Measurement of horizontal and vertical (Tarnow's classification) furcation defects PCPUNC‐15 periodontal probe	Clinical periodontal measurements performed by a periodontics resident and CBCT measurements by an Oral Radiology resident	Significant difference between CBCT and clinical measurements	CBCT can be used as an adjunct to clinical furcation measurements and adds useful diagnostic information to assess trifurcation defects.
Lam et al. (2022) Hong Kong	Cross‐sectional University	No funding	Patients with periodontitis and at least FI Class II *N* = 40 (teeth) Mean age: NR	FI in the horizontal and vertical component	CBCT (2 types) FOV: 100 × 80 mm and 100 × 100 mm Voxel size: 0.125 mm and 0.2 mm Tube voltage: 100 kV and 90 kV Tube current: 5 mA and 8 mA Scanning time: NR Slice thickness: NR	Clinical examination NR Periapical radiographs Measurements according to the modified vertical furcation	A single calibrated examiner for the clinical examination. 15 examiners for the CBCT	Reliability of CBCT measurements among examiners: ICC 0.560 (95% CI 0.446–0.686).	Poor to fair agreement between clinical measures/periapical radiographs and CBCT for subclass of vertical FI diagnosis.
Alotaibi et al. (2024) Saudi Arabia	Cross‐sectional; University	NR	Patients with generalized Stages II–IV periodontiti, Grades B and C (Tonetti et al. 2018) *N* = 25 Mean age: NR	FI (*n* = 68 M) according to Glickman's 1958	CBCT NR	Clinical examination Modified Glickman's classification Nabers probe	NR	Significant differences between the clinical and CBCT measurements of Grade II FI in the buccal region of the first molar and Grade III FI in the buccal region of the second molar and lingual region of the first molar	CBCT may be more accurate in detecting FI.
Studies focusing on alveolar bone crest									
Goodarzi Pour et al. (2015) Iran	Cross‐sectional; Private clinic	NR	Patients seeking dental implant treatment. *N* = 30 Mean age: NR	Bone loss around teeth (*n* = 38)	CBCT FOV: NR Voxel size: 0.3 mm Tube voltage: 80 kV Tube current: 4.3 mA Scanning time: 17 s Slice thickness: NR	Intra‐surgical measurements Distance CEJ‐ AC measured on buccal, lingual/palatal, mesial and distal surfaces	Clinical measurements performed by the surgeon during the surgery. NR for CBCT measurements.	In the buccal lingual/palatal, mesial and distal surfaces no significant difference was observed between the values obtained using CBCT and the surgical method	CBCT enables accurate measurement of bone loss comparable to surgical exploration and can be used for diagnosis of bone defects in periodontal diseases.
Zhang et al. (2018) USA	Cross‐sectional; University	NR	Patients seeking dental treatment *N* = 80 Mean age: 54.9 (SD: 15.4) years	Bone loss	CBCT FOV: 150 × 90 mm Voxel size: 0.2 mm Tube voltage: 90 kV Tube current: 10 mA Scanning time: 16 s Slice thickness: 1 mm	Clinical examination CAL Periapical and/or bite‐wing radiographs CEJ‐AC	NR	Highly significant positive correlations between CBCT and CAL, (*r* = 0.64), relative to perioapical radiographs with CAL (*r* = 0.55)	CBCT is a valuable tool for periodontal assessment.
Yang et al. (2019) China	Cross‐sectional; Hospital	NR	Patients with generalized chronic periodontitis *N* = 13 Mean age: 32.5 (range 22–49) years	Bone loss on 30 teeth (*n* = 180 sites)	CBCT FOV: 100 × 100 mm Voxel size: 0.125 mm Tube voltage: 85 kV Tube current: 4 mA Scanning time: 17.5 s Slice thickness: NR	Clinical examination CAL Intra‐surgical measurements CEJ‐AC PCPUNC 15 probe	Three trained and calibrated periodontists for CBCT measurements.	There were no differences between CBCT and intra‐surgical measurement in 56% of sites, while 37% differed by at most 1 mm, 31% by at most 2 mm	The results of CBCT do not agree with results of intra‐surgical measurement.
Patil et al. (2023) India	Cross‐sectional; Dental college	NR	Patients with chronic periodontitis requiring periodontal surgery *N* = 40 Mean age: 46.25 (SD: 3.5) years	Bone loss (vertical and horizontal) on periodontal defects (*n* = 202)	CBCT FOV: NR Voxel size: 0.125 mm Tube voltage: 90 kV Tube current: 10 mA Scanning time: 13 s Slice thickness: NR	Intra‐surgical measurements CEJ‐AC Periodontal probe	A single, trained and experienced periodontist for the intra‐surgical measurements and a trained and experienced radiologist for the CBCT measurements	The ICC between CBCT and intra‐surgical measurements was optimal (> 0.89) in all sites (buccal, distal, mesial and palatal)	CBCT is a valuable tool that can complement clinical measurements and offer significant diagnostic information for bone defects evaluation.
Fleiner et al. (2024) Germany	Cross‐sectional; University	No funding	Patients with Stage III/IV periodontitis (Tonetti et al. 2018) *N* = 20 Mean age: 54.3 (SD: 10.7) years	Bone loss and FI (*n* = 120 teeth)	CBCT FOV: 80 × 80 mm Voxel size: 0.16 mm Tube voltage: 80 kV Tube current: 12 mA Scanning time: 12.22 s Slice thickness: 1 mm	Clinical examination CAL UNC15 probe Panoramic radiograph CEJ‐AC	One specialized examiner for clinical measures and one specialised investigator for all imaging measures	Compared to clinical measurements, the mean total error of measurement (SD) was larger in the 2D panoramic imaging modality (0.66 [0.48] mm) than in the 3D CBCT evaluation (0.27 [0.08] mm)	Software‐based CBCT analysis may allow accurate assessment of periodontal bone levels.

Abbreviations: AC, alveolar crest; BD, base of the defect; CBCT, cone beam computed tomography; CEJ, cemento‐enamel junction; FI, furcation involvement; FOV, field of view; ICC, intraclass correlation coefficient; NR, not reported; RCT, randomized controlled trial.

**TABLE 4:** Summary of the studies investigating the diagnostic accuracy of fractal analysis on periapical or panoramic radiographs.

Author (year), country	Study design, setting	Funding	Population	Diagnosed condition and definition	Index measure	Reference measure	Test assessor	Test accuracy measure	Main conclusion
Shrout et al. (1998) USA	Cross‐sectional; University	NR	Patients seeking periodontal treatment *N* = 61 Mean age: 41.9 years	Periodontal health/gingivitis (*n* = 29) vs. periodontitis (*n* = 32) based on PD, BOP and CAL	Trabecular bone structure on the mandibular posterior area assessed by FD calculated on periapical radiographs (by ImageFractal)	Clinical examination NR	Experienced periodontitis for the clinical measures	Mean FD For gingivitis: 2.061 For periodontitis: 2.049	Fractal analysis could be used to distinguish between gingivitis and periodontitis patients.
Updike and Nowzari (2008) USA	Cross‐sectional; University	Grant from the University of Southern California Departments of Advanced Periodontics and Advanced Orthodontics	Patients seeking for periodontal treatment *N* = 108 Mean age: 48.2 (SD: 15.9) years	Periodontal health (*n* = 36) vs. moderate periodontitis (*n* = 36) vs. severe periodontitis (*n* = 36) based on CAL	Trabecular bone structure on the mandibular anterior area assessed by FD calculated on periapical radiographs (by ImageJ)	Clinical examination PPD CAL	NR	Mean FD For healthy: 1.74 For moderate periodontitis: 1.66 For severe periodontitis: 1.64 Healthy periodontal patients had significantly higher FD values. The AUC was 0.758	Fractal analysis can differentiate between affected and non‐affected patients and it can be used to improve current diagnostic techniques.
Sener et al. (2015) Turkey	Cross‐sectional; University	NR	Patients seeking dental treatment *N* = 100 Mean age: 41 (SD: 9.2) years	Periodontal health (*n* = 50) vs. moderate periodontitis (*n* = 50) based on 1999 Classification	Trabecular bone structure on the mandibular posterior area assessed by FD calculated on periapical radiographs (by ImageJ)	Clinical examination PPD CAL Williams probe	Single calibrated examiner	Mean FD: For healthy: 1.02 For periodontitis: 0.83	Patients with periodontitis demonstrated lower FD values than patients with healthy periodontium. Fractal analysis can be used for diagnosis and monitoring.
Khajavi et al. (2017) Iran	Cross‐sectional, NR	NR	NR *N* = 40 Mean age range: 37–42 years.	Periodontal health (*n* = 20) vs. moderate periodontitis (*n* = 20) based on 1999 Classification	Trabecular bone structure on the mandibular molar area assessed by FD calculated on periapical radiographs (by ImageJ)	Clinical examination PPD CAL Williams probe	NR	Mean FD: For healthy: 1.01 For periodontitis: 0.84	Fractal analysis can efficiently quantify differences in trabecular pattern of bones between healthy and periodontitis patients.
Aktuna Belgin and Serindere (2020) Turkey	Cross‐sectional; University	No funding	Patients seeking periodontal treatment *N* = 70 Age range: 18–70 years	Periodontal health (*n* = 35) vs. periodontitis (*n* = 35) based on CAL	Trabecular bone structure on mandibular molar area assessed by FD calculated on digital periapical radiographs (by ImageJ)	Clinical examination PPD CAL	Single calibrated examiner	Mean FD: For healthy: 1.04 For periodontitis: 0.97	Fractal analysis can be used to detect periodontal destruction.
Soltani et al. (2021) Iran	Cross‐sectional; Hospital	Isfahan University of Medical Sciences Grant	Patients with different stages of periodontitis *N* = 80 Mean age: 30.8 (SD: 11.9) years	Periodontal health (*n* = 36) vs. periodontitis (*n* = 44) based on CAL	Trabecular bone structure on first mandibular molars assessed by FD calculated on digital periapical radiographs (by ImageJ)	Clinical examination CAL	One operator for the clinical measure and one trained senior dental student for the imaging measure	Mean FD For healthy: 1.63 For mild periodontitis: 1.60 For moderate periodontitis: 1.55 For severe periodontitis: 1.54	Fractal dimension was significantly lower in the surrounding bone of teeth with moderate and severe periodontitis compared with those without attachment loss.
Korkmaz et al. (2023) Turkey	Cross‐sectional; University	NR	Patients seeking periodontal treatment *N* = 61 Mean age: 28.5 (SD: 4) years	Periodontal health (*n* = 33) vs. Stage III/IV, Grade C periodontitis (*n* = 28) based on WWP 2018 Classification	Trabecular bone structure on first mandibular molar assessed by FD calculated on panoramic radiograph (by ImageJ)	Clinical examination PPD CAL	One senior periodontitis for the clinical measure and one experienced radiologist for the imaging measure	Mean FD For healthy: 1.51 For Stage III/IV periodontitis: 1.37	Fractal analysis can effectively detect trabecular microarchitectural differences in patients with periodontitis compared to periodontally healthy individuals.
Mishra et al. (2023) India	Cross‐sectional; University	No funding	Patients seeking periodontal treatment *N* = 75 Age range: 25–60 years	Periodontal health (*n* = 15), Stage I (*n* = 15), Stage II (*n* = 15), Stage III (*n* = 15), and Stage IV (*n* = 15) periodontitis based on WWP 2018 Classification	Trabecular bone structure around the tooth with the greatest CAL assessed by FD calculated on periapical radiographs (by ImageJ)	Clinical examination PPD CAL	Single calibrated examiner	Mean FD For healthy: 1.21 For Stage I periodontitis: 1.21 For Stage II periodontitis: 1.19 For Stage III periodontitis: 1.11 For Stage IV periodontitis: 1.02 FD ≤ 1.18 predicts periodontitis (all stages) with a sensitivity: 78.3 and specificity: 80 and AUC: 0.77	FD decrease with the stage of periodontitis, with the highest value in healthy controls and the lowest in Stage IV periodontitis.
Yarkac et al. (2023) Turkey	Cross‐sectional; University	No funding	Patients seeking periodontal treatment *N* = 200 Mean age range: 30–45 years	Periodontal health (*n* = 50), Stage I (*n* = 50), Stage II (*n* = 50), and Stage III (*n* = 50) periodontitis based on WWP 2018 Classification	Trabecular bone structure on first mandibular molar assessed by FD calculated on panoramic radiograph (by ImageJ)	Clinical examination PPD CAL	Clinical measures by a single calibrated clinician and imaging measure by an experienced examiner	Mean FD: For healthy: 1.44 For Stage I: 1.36 For Stage II: 1.35 For Stage III: 1.28. FD ≤ 1.409 predicts periodontitis with sensitivity: 74% specificity: 74% AUC: 0.828	Mean FD value was significantly higher in the healthy group.
Eser and Saribaş (2024) Turkey	Cross‐sectional; University	NR	Patients seeking for periodontal treatment *N* = 128 Mean age: 33.9 (SD: 9.6) years	Gingivitis (*n* = 64) and periodontitis (*n* = 64)	Trabecular bone structure on first mandibular molar assessed by FD calculated on panoramic radiograph (by ImageJ)	Clinical examination NR	NR	Mean FD For gingivitis: 1.195 For periodontitis: 1.196	No significant differences in fractal dimension between gingivitis and periodontitis.

Abbreviations: CAL, clinical attachment level; FD, fractal dimension; NR, not reported; PPD, probing pocket depth.

**TABLE 5:** Summary of the studies investigating the diagnostic accuracy of digital methods to analyse radiographic imaging.

Author (year), country	Study design, setting	Funding	Population	Diagnosed condition and definition	Index measure	Reference measure	Test assessor	Test accuracy measure	Main conclusion
Computer‐assisted densitometric image analysis (CADIA) for the assessment of alveolar bone density									
Payne et al. (2013) USA	Prospective cohort; University	National Institute of Dental and Craniofacial Research, SDD and placebo provided by CollaGenex Pharmaceuticals (now Galderma)	Post‐menopausal women with osteopenia and periodontitis *N* = 117 Age range: 45–70 years	Moderate to severe periodontitis	Vertical bitewing and CADIA	Clinical examination RCAL (Florida disk probe) PPD (UNC 15 probe)	One examiner for radiographic measurements; clinical measurements by trained personnel	Reproducibility of CADIA measurements: ± < 2 mm (99.6%); ± < 1 mm (95.8%); Association between ABD and ABH at 1 year: Significant (OR = 3.15, 95% CI: 1.84–5.39, *p* < 0.0001); Not maintained at 2 years	CADIA was able to identify concurrent changes in bone density and height after 1 year, but this correlation did not continue over the 2‐year period.
Deas et al. (1991) USA	Prospective cohort; University	No funding	Patients with untreated periodontitis *N* = 21	Periodontitis	Standardized Rx (vertical bitet wing) and CADIA	Clinical examination, duplicate PAL	One calibrated examiner	Mean density loss for complexes with probing attachment loss ≥ 2 mm: −6.10 CADIA units, mean density loss for complexes without significant probing attachment loss: −2.36 CADIA units	Sites with initial probing depths > 3 mm are more likely to show disease progression. Density changes in radiographs did not reliably predict probing attachment loss.
Digital subtraction									
Reddy et al. (2000) USA	Prospective cohort; University	NIH NIDCR grant	Patients with moderate to advanced adult periodontitis *N* = 44 Mean age 45.8 years	Moderate to advanced adult periodontitis; radiographic evidence of 30%–50% bone loss, pockets ≥ 4 mm, > 4 mm attachment loss	Standardized vertical bite wing; Substraction radiography	Clinical examination CAL (occlual stent, WHO probe), BOP, GI, PI, PPD	One calibrated examiner	Predictive models Baseline Model: Sensitivity of 61.8% and specificity of 68.5%. 6‐Month Data Model: Sensitivity increased to 70.9% and specificity to 83.3%. Model with Digital Subtraction Radiography: Sensitivity of 80.0% and specificity of 93.9%	Digital subtraction radiography effectively predicts future attachment loss; short‐term bone and attachment loss are strong indicators of long‐term disease progression.
Preshaw et al. (1999) UK	Prospective cohort; NR	No funding	Adults with untreated chronic periodontal disease Subgroups: non‐smokers, ex‐smokers, current smokers *N* = 41 Mean age: 43 years (range 30–64)	Untreated moderately advanced chronic adult periodontitis	Standardized vertical bite‐wing radiographs were exposed at test sites and analysed with Digital Subtraction Analysis	Clinical examination (PI), PD, BOP, and relative clinical AL	One calibrated examiner	NR	Subtraction radiography detected small, statistically significant bone loss before treatment, but post treatment, it showed a reversal with bone gain. While changes were statistically significant, they were not clinically significant.
Eickholz et al. (1998) Germany	Prospective cohort; University	No funding	Patients with severe periodontitis *N* = 24 Age range: 32–63 years	Interproximal intrabony defect	Standardized bitewing with digital subtraction analysis and CADIA	Clinical examination GI, PPD, PAL‐V, PlI UNC 15 probe	One calibrated examiner	Specificity: not explicitly detailed, but high variability in radiograph suitability indicates challenges in maintaining consistent quality for accurate assessment	Subtraction radiography d etected significant bony fill and bone gain after GTR therapy, with small but significant correlations between bone gain and CADIA‐value. Vertical attachment gain did not correlate with bony fill.
Eickholz et al. (1997) Germany	Prospective cohort; University	No funding	Patients with severe periodontal disease *N* = 21 Age range: 25–61 years	FI Grades II and III	Standardized vertical bite wing with digital subtraction analysis	Clinical examination GI, PPD, CAL‐V, CAL‐H, PlI UNC 15 probe	One calibrated examiner	NR	Subtraction Radiography is effective for assessing bony regeneration, but the technique's high sensitivity to projection geometry and anatomical factors resulted in a large number of radiographs being unsuitable for analysis.
Brägger et al. (1992) Switzerland	Prospective cohort; University	Swiss National foundation	Patients with intrabony defect and furcation involvement *N* = 25	Intrabony defect and FI	Standardized vertical bitewing with digital subtraction analysis and CADIA	Clinical examination Rec, CAL, PPD, PI, Gi Michigan Probe Intra‐surgical bony defect measures CEJ‐ABL and H	One calibrated examiner	NR	Radiographic density changes and clinical parameters showed low correlation, indicating that they provide distinct yet complementary insights into periodontal healing and bone regeneration.
Jeffcoat (1992) USA	Controlled clinical trial; NR	NIH Grant	Patients with evidence of alveolar bone loss (no treatment) *N* = 38 NR	Periodontitis	Digital subtraction radiography	Clinical examination CAL Automated periodontal probe	NR	Sensitivity: 71.6% ± 1.0% Specificity: 80.4% ± 0.6%	When precise methods are used, electronic probing and digital subtraction radiography show high concordance in detecting periodontal disease. Discrepancies may arise from false positives/negatives or differing disease manifestations.

Abbreviations: CAL, clinical attachment level; FI, furcation involvement; NR, not reported.

**TABLE 6:** Summary of the studies investigating the diagnostic accuracy of non‐ionizing‐radiation methods.

Author (year), country	Study design, setting	Funding	Population	Diagnosed condition and definition	Index measure	Reference measure	Test assessor	Test accuracy measure	Main conclusion
Intraoral ultrasonography									
Tanaka et al. (2023) Hong Kong	Cross‐sectional; Hospital	NR	Patients seeking periodontal treatment *N* = 35 NR	FI on mandibular molars (*n* = 61) classified according to Hamp et al. (1975)	USG B‐mode imaging with a 18 MHz high‐frequency hockey stick linear USG probe.	Clinical examination Nabers probe Periapical radiographs, CBCT	One practitioner for probing and periapical radiographs. For USG, a single experienced radiologist blind to clinical findings	Kappa value for agreement of FI between USG and CBCT: 0.792. Compared to CBCT, USG accuracy: 98.4%; Sensibility: 98.3% Specificity: 100%	USG may be a reliable diagnostic tool for assessment of FI of mandibular molars with a similar performance to CBCT.
Magnetic resonance imaging									
Probst et al. (2021) Germany	Case–control; University	In‐house funds from the Department of Neuroradiology, Technical University of Munich	Patients with periodontal disease (*N* = 42), Mean age: 56 years (SD: 14.6), Age range: 28–79 years. Healthy control subjects (*N* = 34), Mean age: 23 years (SD: 1.9), Age range: 21–32 years	Periodontal disease diagnosed by clinical attachment loss in ≥ 2 interdental sites at non‐adjacent teeth and/or probing depth > 3 mm.	MRI imaging sequence: T1‐ and T2‐weighted Type of scanner: 3‐T whole‐body MR scanner Contrast agent: None	Clinical examination CAL OPT radiography	One experienced neuroradiologist and one experienced radiologist/dentist	ICC for image quality evaluation: 0.875. Significant differences in osseous edema based on pocket depth	MRI identifies intraosseous changes associated with periodontitis, aiding in early diagnosis and disease monitoring.
Juerchott et al. (2020), Juerchott et al. (2020) Germany	Prospective cohort; University	ITI Foundation, Switzerland, Grant	Patients with Stages III and IV periodontitis *N* = 23 Mean age: 52.9 (SD: 10.6) years	FI on maxillary molars (*n* = 29 first and *n* = 36 s maxillary molars for a total of 195 furcation entrances) classified according to Hamp et al. (1975) and Tarnow and Fletcher (1984)	MRI Imaging sequence: T1‐weighted VIBE Type of scanner: 3 Tesla MRI scanner Contrast agent: None Acquisition time: 6.03 + 5.31 min	Clinical examination PPD, PAL six sites per tooth Periodontal probe Horizontal FI component measured at three sites (buccal, mesio‐palatal and disto‐palatal) PQ2N Nabers probe CBCT	Clinical measurements by two experienced and calibrated dentists. CBCT and MRI by two independent observers blind to clinical evaluation	Excellent intra‐ and inter‐rater agreement for both CBCT and MRI. High agreement between CBCT and MRI for both horizontal and vertical measurements of furcation defects. For the detection of furcation defects, MRI achieved a sensitivity: 98% and specificity: 100% for horizontal and 99%/99% for vertical bone loss. Sensitivity of MRI for FI classification: For degree I: 88% For degree II: 89% For degree III: 100% For subclass A: 95% For subclass B: 91% For subclass C: 80% Compared to clinical diagnosis, MRI showed a sensitivity of 62.8% and a specificity of 84.6% for the detection of FI (calculated values)	Non‐ionizing, non‐contrast‐enhanced dental MRI has demonstrated high reliability and high levels of agreement with CBCT in determining horizontal and vertical furcation defects in maxillary molars.
Ruetters et al. (2019) Germany	Prospective feasibility study; University	NR	Patients with periodontitis according to Armitage 1999 classification system *N* = 5 Mean age: 57.8 (range: 34–68) years	Bone loss around teeth (*n* = 21). Measures taken on 41 sites	MRI Imaging sequence: T2‐ and T1‐weighted Turbo Spin Echo Sequences. Contrast agent: GBCA (Dotarem) Acquisition time: 20 min	Periapical radiograph Distance root apex—deepest bony defect compared to distance root apex—pulp horn, with the latter chosen as a consistent reference, unlike alternatives like the CEJ	Two raters, one dentist and one radiologist, each of them with at least 4 years of clinical experience	For measurements of the ratio of periodontal bone loss, defined as the distance between the root apex to the bottom of the bony defect, divided by the distance between the root apex to the top of the pulp horn, there is a strong correlation between MRI and periapical radiographs. DMRI showed high reproducibility and reliability	The findings of the present study indicate excellent agreement of MRI and periapical radiographs when measuring residual periodontal bone support.

Abbreviations: CBCT, cone‐beam computed tomography; FI, furcation involvement; GBCA, gadolinium‐based contrast agent; MRI, magnetic resonance imaging; NR, not reported; PAL, probing attachment level; USG, ultrasonography; VIBE, volumetric interpolated breath‐hold examination.

### 3.4 Quantitative Synthesis

**FIGURE 3:**
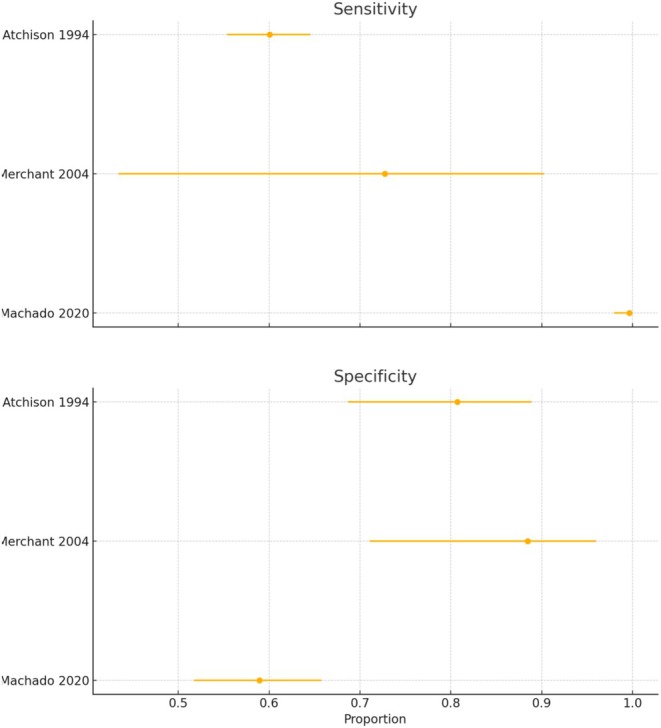
Pooled sensitivity and specificity across the studies (PICO 1).

**FIGURE 4:**
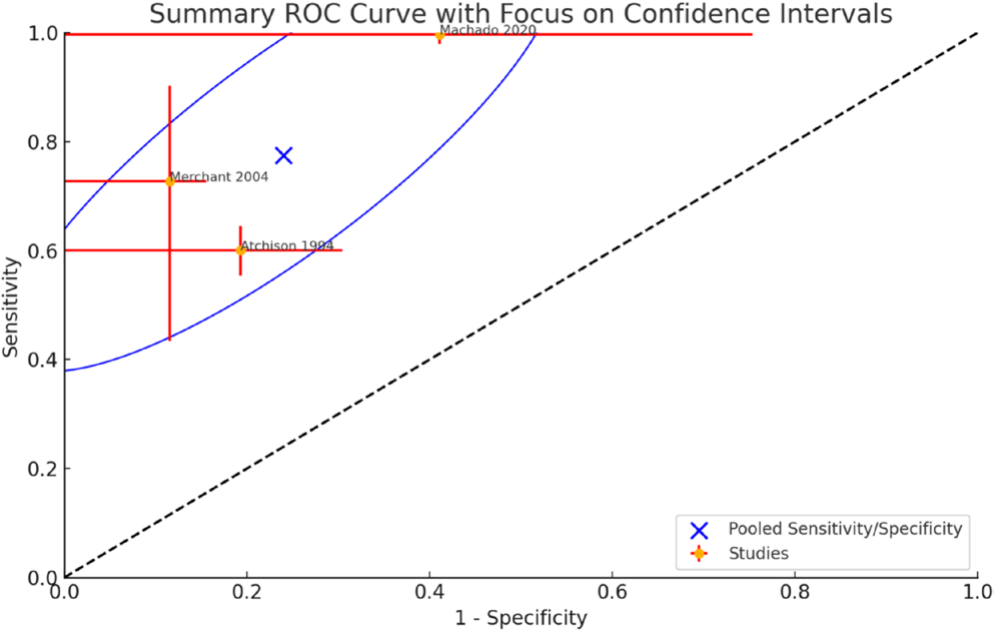
Summary ROC (SROC) curve incorporating individual study estimates (PICO 1).

For PICO 1, pooled data analysis was based on three studies comprising 983 patients (Atchison et al. 1995; Machado et al. 2020; Merchant et al. 2004). The pooled sensitivity was 0.77 (95% CI: 0.66–0.85), while the pooled specificity was 0.76 (95% CI: 0.64–0.84) with a high level of accuracy (0.82). The DOR was 137.99 (95% CI: 6.99–368.90), indicating strong diagnostic accuracy. Heterogeneity was moderate to high, with *I* ^2^ values of 74.9% for sensitivity and 81.3% for specificity, suggesting some variability in diagnostic performance across the studies. The SROC curve shows the balance between sensitivity and specificity, with CIs around individual studies and pooled estimates reflecting variability in the diagnostic test performance (Figures 3 and 4).

**FIGURE 5:**
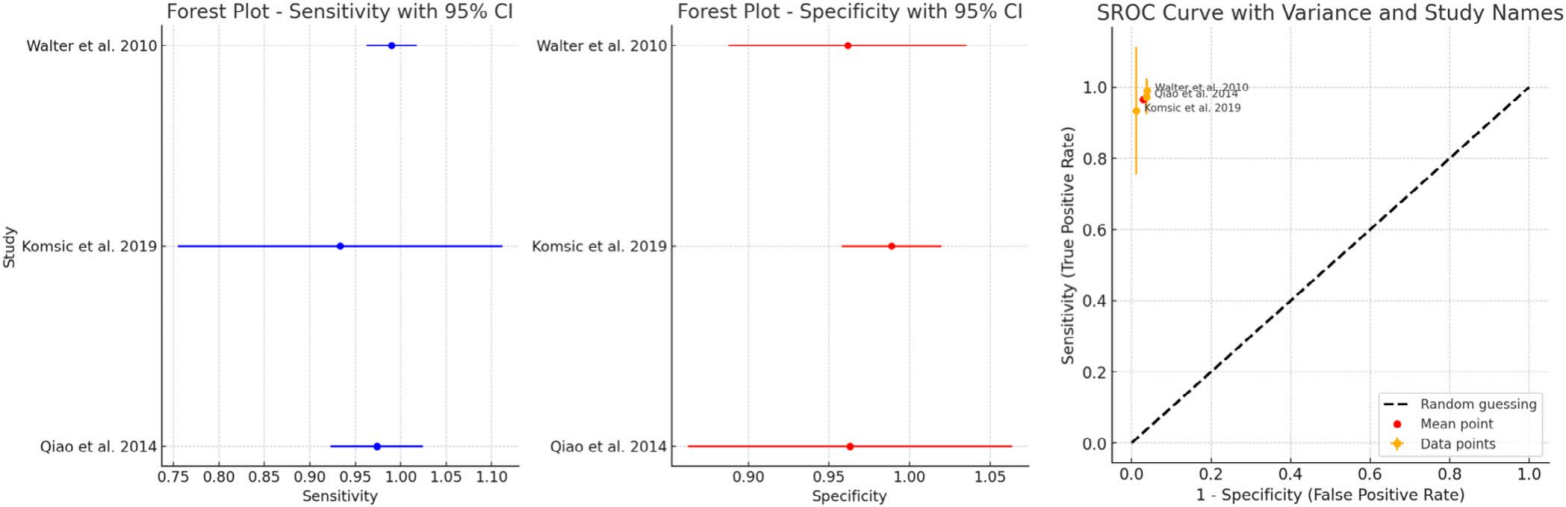
Pooled data analysis and summary ROC (SROC) curve for studies comparing CBCT and Intra‐surgical measurements as gold standard (PICO 2).

For PICO 2, meta‐analysis was deemed possible only for studies assessing the diagnostic accuracy of CBCT for furcation involvement (FI). Only 3 studies (Komšic et al. 2019; Qiao et al. 2014; Walter et al. 2010) out of the 14 assessing the role of CBCT in FI were sufficiently homogenous in design to be pooled together. These three studies analysed FI in maxillary first and second molars (*n* = 168) and employed intra‐surgical measures as a gold standard reference. FI was assessed in terms of presence/absence (yes/no), distinguishing between Grades 2 and 3 (yes) versus Grades 0 and 1 (no). Thus, compared to those of direct intra‐surgical measurements, the pooled sensitivity and specificity of CBCT to correctly diagnose/detect an FI were 0.98 (95% CI: 0.96–1.00) and 0.98 (95% CI: 0.95–1), respectively (*I* ^2^: 0%), with a pooled DOR of 1450.14 (95% CI: 181.38–11593.50), indicating that CBCT discriminates correctly the presence of FI in maxillary molars (Figure 5). Indeed, the calculated diagnostic accuracy for CBCT for FI was 0.99. Conversely, when compared to clinical measurements to diagnose FI (Walter et al. 2009; Cimbaljevic et al. 2015; Zhu and Ouyang 2016), CBCT was associated with excellent sensitivity (0.95; 95% CI: 0.91–0.98) but very low specificity (0.12; 95% CI: 0.06–0.17), with a pooled DOR of 0.20 (95% CI: 0.07–0.57) and very high heterogeneity (*I* ^2^: 89.14%; Figure S1).

### 3.5 Risk of Bias and Quality Assessment

**FIGURE 6:**
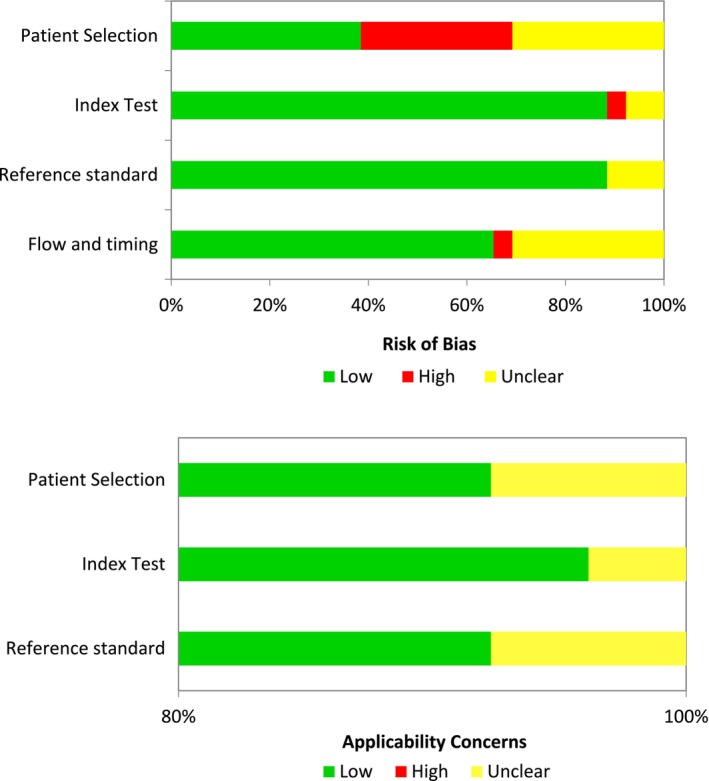
Risk of bias and applicability concerns graph (PICO 1).

The risk of bias and applicability assessment of the studies selected for the PICO 1 are presented in Figures 6 and S4.

In the patient selection domain, 28% of the articles were considered to have an unclear risk of bias, due to unspecified methods of patient selection and the absence of clear exclusion criteria. Eight studies were judged to have a high risk, due to the employment of purposive sampling. One study was rated at high risk in domain 2, because the threshold used for the index tests and the blinding of examiners were not specified (Saberi et al. 2017). Unclear concerns regarding applicability in domain 3 were identified in 2 of the 17 selected studies because of the use of CPITN (community periodontal index treatment need) as the reference standard and the lack of sufficient details on the clinical measurements (Douglass et al. 1986; Walsh et al. 1997).

**FIGURE 7:**
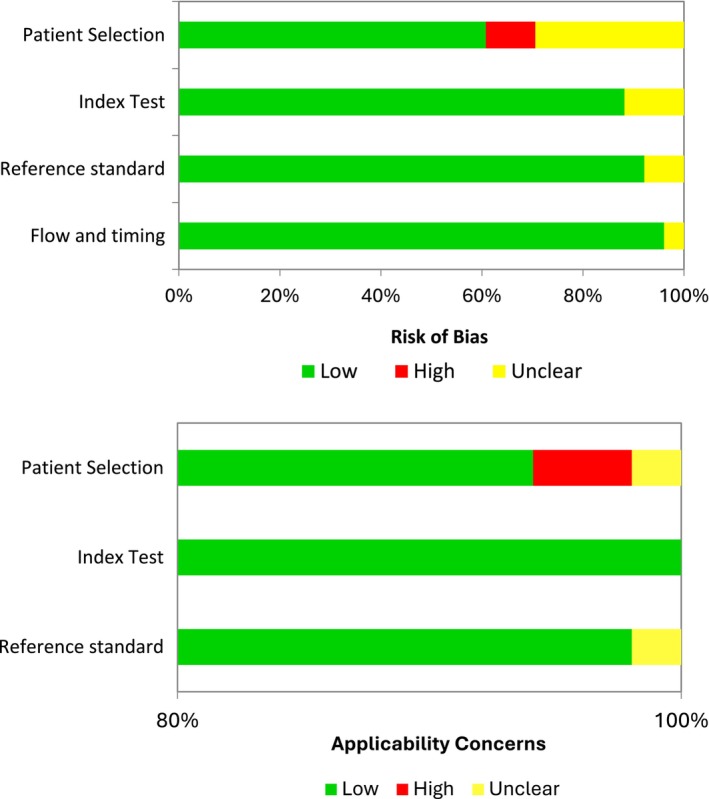
Risk of bias and applicability concerns graph (PICO 2).

The risk of bias of the 51 studies included in PICO 2 is summarized in Figures 7 and S5. In domain 1, five studies were classified at high risk of bias, while 29% were considered to have an unclear risk, due to the use of case–control designs, the absence of consecutive or random sampling or the selection of specific patient populations. In the index test domain, six articles showed an unclear risk due to the lack of information regarding the blinding of examiners or the threshold used. The absence of information about patient characteristics (de Faria Vasconcelos et al. 2012) and the study focus on implant treatment rather than on periodontal disease (Goodarzi Pour et al. 2015) resulted in high applicability concerns in two studies and unclear in one (Shrout et al. 1998).

### 3.6 Risk of Bias Across Studies

No significant publication bias was observed (Deeks et al. 2005) based on DOR when considering studies reporting the accuracy of standard 2D radiography (Figure S2) and CBCT (Figure S3).

## 4 Discussion

The present systematic review provides an updated synthesis of the body of evidence supporting the role of imaging in the diagnosis of periodontitis. The results are based on two focus questions, which retrieved a total of 69 publications. A meta‐analysis was performed for the diagnostic accuracy of standard 2D radiography for the diagnosis of periodontitis at the patient level and for the diagnostic accuracy of CBCT for furcation defects on maxillary molars. The great variability in study designs, population characteristics, reference measure and disease definitions demands caution at the time of interpreting the results.

### 4.1 Standard 2D Radiography

PICO 1 provided answer to the need for assessing the diagnostic accuracy of standard 2D radiography for the diagnosis of periodontitis. The quantitative pooled data analysis highlights a high level of diagnostic accuracy in terms of DOR, sensitivity and specificity, supporting the principle that clinical and radiographic diagnosis can be considered as complementary. In fact, even though the identification of a ‘periodontitis case’ is primarily based on clinical findings (Tonetti et al. 2018) according to the 2018 WWP Classification System, radiography plays a complementary role in the case of staging (when CALs cannot be assessed). Additionally, in establishing the grade, radiographs provide indirect evidence, but it is an essential evaluation tool to assess the risk of disease progression (Caton et al. 2018; Machado et al. 2020; Jacobs et al. 2024). Radiography finds its applicability in the prognosis and longitudinal evaluation of the risk of disease recurrence (Lang and Bartold 2018; Papapanou et al. 2018).

Beyond the inherent limitations of radiography (e.g., incomplete view of the bucco‐lingual aspects of the interproximal bone, underestimation of initial alveolar bone loss, overestimation of severe bone loss), the lack of reporting about standardization is noteworthy (Table 1). The accuracy and reliability of panoramic and intraoral radiography are indeed greatly influenced by the standardization of the alignment among radiographic components, such as the image receptor plane, the X‐ray beam and the teeth (Åkesson, et al. 1989a, 1989b; Benn 1990; Eickholz and Hausmann 2000; Pepelassi et al. 2000).

The diagnostic accuracy of 2D radiographs was also explored at the site level considering both clinical and intra‐surgical measurements as reference standards (Table 2). However, due to heterogeneity of the selected studies, no quantitative synthesis could be performed. Moreover, the correlation between clinical measures and radiographic methods for furcation evaluation resulted in high heterogeneity, with sensitivity values ranging from 30% to 70% and specificity values ranging from 70% to > 90%. A general tendency to underestimate the lesions was observed, and all methods became more reliable in case of more advanced level of involvement (i.e., Grades II and III furcation). While assessing bone crater morphology is essential, none of the included studies directly measured this aspect. Some studies indirectly evaluated bone morphology by distinguishing between vertical and horizontal components, but a detailed analysis of bone craters was not their focus. There are several parameters, such as the anatomical complexity of molars, the different locations of the tooth, the site of furcation entrance and experience of the clinical examiner, that could have influenced the magnitude of the correlation (Graetz et al. 2014; Komšic et al. 2019; Jolivet et al. 2022).

The results for the relationship between radiographic bone level, intrabony defects and CAL account for both moderate to weak correlation and low sensitivity with higher accuracy for bitewing radiography. Tooth type (maxillary molars and mandibular incisors less reliable), timing of evaluation and the index measure applied might have influenced the results. Previous seminal short‐term retrospective studies have already addressed the reasons for this low diagnostic correlation (Armitage et al. 1994; Goodson et al. 1984; Mann et al. 1985). The measurement error associated with either technique or the analysed tissue target might account for a poor site correlation. In fact, the changes detected by radiography relate with the bone height, while the changes detected by the clinical measure relate with both anatomical attachment level and tissue tone. Moreover, the soft‐ and hard‐tissue remodelling could occur in distinct time, thus progressing independently (Goodson et al. 1984; Machtei et al. 1997; Teles et al. 2018).

### 4.2 CBCT

CBCT has been used for more than two decades, and over 250 different CBCT models are available on the market (Gaêta‐Araujo et al. 2020). This is deductible from the different protocols of image acquisition detailed in the selected studies, including differences in the field of view, peak voltage, voxel size, scanning time and slice thickness. This highlights a very important point: the image acquisition protocol as well as the subsequent image evaluation is influenced by the technical characteristics of the CBCT and those set by the practitioner. Generally, these should be adapted to obtain an adequate image quality while minimizing the radiation dose to which patients are exposed. Still, it remains an ionizing‐radiation‐based technique whose use must be justified by clear diagnostic advantages.

CBCT provides a 3D image of the dental and periodontal tissues without the limitation of superposing anatomical structures that can hamper the correct interpretation of the standard 2D radiographs. It is relatively rapid to perform, and several pieces of information supporting diagnosis and treatment planning can be gathered. Back in 2017, the American Academy of Periodontology published a consensus statement indicating that there was little evidence to support CBCT as a routine replacement or adjunct to 2D imaging in the management of periodontitis (Mandelaris et al. 2017). Based on expert opinions, CBCT was considered useful in case of severe periodontitis, when furcation areas are involved, to evaluate other anatomical structures such as the maxillary sinus or the inferior alveolar nerve and when planning for dental implants. In these clinical scenarios, CBCT may be more appropriate than 2D radiographs, but no evidence for this was provided (Mandelaris et al. 2017). In the present systematic review, 29 studies were considered. CBCT was mainly used to evaluate and classify intrabony defects or FI, and in several studies the diagnostic accuracy of CBCT was compared with intra‐surgical measurements as the reference standard. In general, the studies assessing intrabony defects consistently found that CBCT is a precise, reliable and accurate tool, superior to intraoral radiographs (Grimard et al. 2009). In the case of FI, pooled data analysis showed that the diagnostic accuracy of CBCT to correctly detect FI was very high (0.99), with pooled sensitivity and specificity of 98%, compared to intra‐surgical measurements. Conversely, the sensitivity and specificity changed if clinical measurements were taken as reference. In general, a low level of agreement was found between CBCT classification and clinical classification of furcation defects, particularly for Grade I. This can be most likely explained by the fact that the clinical diagnosis of FI is challenging, and correctly probing this area is not always possible. Frequently, the inability to fully penetrate the furcation with the probe, even in the absence of clinical attachment, contributes to the evidenced disparity between clinical and CBCT measurements. When the tip of the probe does not penetrate the furcation, Grade III FI can be assumed if the sum of the vestibular and lingual probing depths exceeds the tooth's vestibulo‐oral dimension (Graetz et al. 2014; Ammons and Harrington 2006). This highlights the meaning of clinical probing (CAL) and the inherent limitations of probing furcation areas, which has led many studies to consider CBCT as a more reliable reference standard over clinical measurements. Indeed, also other selected studies assessing FI by CBCT (but not included in the pooled data analysis) consistently reported great accuracy in detecting FI and in correctly classifying the degree of horizontal and vertical bone loss (Walter et al. 2009; Cimbaljevic et al. 2015; Pajnigara et al. 2016; Marinescu et al. 2014; Padmanabhan et al. 2017; Zhu and Ouyang 2016; Zhang et al. 2018; Lam et al. 2022; Alsakr et al. 2022; Yusof et al. 2021; Alotaibi et al. 2024). This increased accuracy may also impact treatment planning and therapeutic decision. Indeed, a study by Walter et al. (2012) observed that CBCT assessments of maxillary molars with FI can influence the treatment choice and finally reduce the treatment‐associated costs and time. Thus, several studies concluded that CBCT may be considered as the reference standard in cases of FI and severe periodontal lesions that merit a 3D imaging assessment. However, CBCT imaging can be drastically compromised by artefacts if metallic materials, such as titanium or zirconia implants or restorations, are present in the area of interest. Considering these limitations, its use should be considered on a case‐by‐case basis by weighing the risk/benefit and costs/benefit ratios (Jacobs and Quirynen 2014; Jacobs et al. 2018).

### 4.3 Fractal Analysis

Fractal analysis consists in a mathematical method of analysing standard 2D digital radiographs (periapical or panoramic) to evaluate the morphological pattern of the alveolar trabecular bone and its possible change over time, making this tool interesting to differentiate between health and disease and to monitor response to treatments. A quantitative measure of image complexity is estimated as the fractal dimension (FD); an increase in FD value is observed when the imaging patterns have a more complex structure, while lower values indicate a simpler structure. The selected studies applied the protocol developed by White and Rudolph, which generates FD values representing the morphological features of the bone architecture (White and Rudolph 1999). Almost all the studies used ImageJ software to calculate the fractal size, and thus they can be considered quite homogenous in their methods. In general, these studies found that FD decreases with the severity/stage of periodontitis, with the highest values found in healthy controls (Khajavi et al. 2017; Sener et al. 2015; Updike and Nowzari 2008; Soltani et al. 2021; Korkmaz et al. 2023; Mishra et al. 2023; Yarkac et al. 2023; Eser and Saribaş 2024). Although a standardized cut‐off value of FD remains to be identified, these findings suggest the diagnostic utility of fractal analysis to detect periodontal breakdown, also in the early stages, and to monitor trabecular changes over time. However, it is worth mentioning that 90% of the studies were found to have used an inappropriate method of patient selection and an unclear, thus hardly reproducible, strategy of application and interpretation of the index test in use, consequently affecting the applicability of the results.

### 4.4 USG

USG was introduced as a promising 3D tool to evaluate the morphology of the periodontal tissues by using a high‐resolution, non‐ionizing, miniaturized probe connected to an ultrasound scanner that allows the real‐time visualization of the tooth crown, root, gingival margin and alveolar bone (Mahmoud et al. 2010; Qi et al. 2023). Intraoral USG may offer several advantages over ionizing techniques, particularly for its non‐invasiveness and being less affected by metal artefacts (Müller et al. 2000; Shah 2014). Several studies have shown that USG is accurate in the detection and visualization of periodontal anatomical structures and characteristics, such as gingival margin, gingival/mucosal thickness, interdental papilla height, periodontal pocket, alveolar bone loss and bony defects (Tattan et al. 2020). Most of these measurements were validated against a clinical assessment or a CBCT, with a moderate to good agreement (Tattan et al. 2020). However, most of these in vivo studies applied USG in healthy individuals without periodontal diseases (Salmon and Le Denmat 2012; Zimbran et al. 2013; Tattan et al. 2020; Moore et al. 2022); nor did they assess the diagnostic accuracy of USG for the diagnosis of periodontitis. Indeed, no study was found concerning the diagnosis of the disease, and only one study (Tanaka et al. 2023) met the inclusion criteria of the present systematic review as investigating the role of USG for the diagnosis of FI. In the study by Tanaka et al. (2023), USG showed excellent agreement with CBCT and high diagnostic accuracy (sensibility: 98.3%; specificity: 100%) in the detection of FI and the assessment of the horizontal component of defect involving the furcation area in mandibular molars. Moreover, both CBCT and USG appeared to be more accurate than clinical examination (probing), particularly in case of FI Grade I (Tanaka et al. 2023). However, as stated by the authors, the application of USG may be limited by several factors, such as the USG probe size and design, the narrow field of view, the accessibility of the area scanned (only buccal furcation of mandibular molars were evaluated) and, finally, the operator's skills and experience (Tanaka et al. 2023). Thus, although promising, the routine application of intraoral USG in daily practice will remain under validation until further studies prove that its use for periodontitis diagnosis can be considered reliable, affordable, streamlined and with a positive cost/benefit ratio.

### 4.5 MRI

MRI also is a non‐ionizing technique providing a tomograph or sectional image that is particularly adapted for soft‐tissue evaluation. High‐resolution images can be constructed in all planes, and the use of contrast agents (such as the intravenous gadolinium‐based contrast agent, GBCA) can enhance the diagnostic value of the technique (Juerchott et al. 2018; Lizio et al. 2018). For periodontal diagnosis, only three studies published between 2019 and 2021 were identified (Juerchott et al. 2020b; Probst et al. 2021; Ruetters et al. 2019). Reutters et al. assessed the diagnostic accuracy of MRI in measuring bone loss around teeth in comparison with standard periapical radiographs and reported a very high concordance between the two techniques, together with an excellent inter‐rater and intra‐rater agreement. Indeed, the extent of periodontal bone loss (expressed as a ratio) measured on MRI was 0.0019–0.0056 (depending on the rater), which was smaller than on a periapical radiograph, suggesting good reliability and reproducibility (Ruetters et al. 2019).

With the purpose of detecting and classify FI, Juerchott et al., evaluated the diagnostic accuracy of MRI in 195 furcation entrances of 65 maxillary molars in 23 patients with Stage III or IV periodontitis. The study also reported the clinical classification and CBCT evaluation of the FI. MRI, performed without any contrast agent, showed values of sensitivity and specificity ≥ 98% in detecting inter‐radicular bone loss, and it was highly sensitive in the classification of horizontal grades and vertical subclasses of FI compared to CBCT. Conversely, its sensitivity and specificity were low (62.8% and 84.6%, respectively) when compared to clinical diagnosis. This highlights the value of clinical probing (CAL) and the inherent limitations of probing furcation areas, which has led many studies to consider CBCT as a more reliable reference standard over clinical measurements (Juerchott et al. 2020; Juerchott et al. 2020). Although not meeting the eligibility criteria of the present systematic review, Juerchott et al. (2020) and Juerchott et al. (2020) also evaluated the accuracy of dental MRI with a contrast agent against CBCT as reference standard. In a sample of patients diagnosed with Stages III and IV periodontitis, the authors analysed 192 furcation entrances on MRI T1‐weighted imaging (obtained after the intravenous administration of 0.1 mmol/kg gadoterate meglumine) and compared them with those obtained by CBCT. Their results showed excellent intra‐ and inter‐reader agreement (> 0.90) for both MRI‐ and CBCT‐based detection and classification of FI, with no significant difference for the absolute measurement values of the horizontal component of defect in the furcation area. Compared to that of CBCT, the diagnostic accuracy of MRI for FI was considered excellent, with a sensitivity of 98% and a specificity of 99% (Juerchott et al. 2020; Juerchott et al. 2020). Dental MRI also showed a high accuracy for the three different furcation sites, with sensitivity rates (based on correct identification of presence and grade of FI) of 86% for buccal, 93% for disto‐palatal and 100% for mesio‐palatal FI, differences that the authors explained as due to the relationship between spatial resolution of the applied MRI technique and the width of furcation entrances (Juerchott et al. 2020).

Finally, in the study by Probst et al., MRI was used to discriminate between periodontal health and disease. MRI findings, particularly osseous oedema, correlated with standard clinical periodontal measures (e.g., PPR, BOP), suggesting that MRI could be applied to depict osseous changes with oedema as surrogate marker for early stages of periodontal disease that would not be detected in a standard 2D periapical or panoramic radiograph (Probst et al. 2021).

Globally, MRI—both with and without the use of contrast agents—appeared as a promising imaging modality for the diagnosis of FI and to detect alveolar bone loss in patients with periodontitis. However, several limitations of the techniques must be acknowledged, including the time consumed in acquisition and sequencing, high cost and limited availability. In addition, high resolution is needed to detect the alveolar bone topography and provide detailed information susceptible to contribute to the clinical diagnosis, but artefacts may occur depending on the type of metal/material of dental restorations present in the area of interest (e.g., stainless steel) (Chockattu et al. 2018). It must be noted that in the included studies, the MRI acquisition protocols were different, as were the targeted condition and disease definition. Thus, no robust conclusion could be drawn, and further investigations are awaited to explore the applicability and cost effectiveness of MRI for periodontal diagnosis.

### 4.6 Other Techniques

It is worth noting that no study dealing with other non‐ionizing techniques applied as diagnostic tools for periodontitis met the selection criteria. Thus, their role in assisting clinicians during the diagnostic process remains to be determined. For instance, optical coherence tomography, periodontal endoscopy and fluorescence spectroscopy have been mainly tested to detect subgingival calculus and verify its adequate removal after periodontal treatment (Buchalla et al. 2004; Chang et al. 2023). Although it is essential to correctly evaluate the location and amount of calculus in patients with periodontitis, this is not a diagnostic criterion and thus the diagnostic performance, in terms of accuracy and reliability, of these techniques cannot be estimated. Moreover, these techniques are usually characterized by sophisticated hardware components, expensive disposable materials and a steep learning curve for the operator, both in terms of correct applications and imaging interpretation, which can be highly influenced by the presence of blood, saliva, debris and water.

Finally, it must be acknowledged that none of the selected studies investigated the safety, applicability and cost effectiveness of these diagnostic tools. These important aspects, which clearly influence the implementation of a new technology, remain to be explored.

### 4.7 Implications for Future Research

- The evaluation of radiographic techniques should be tested or compared against the clinical diagnostic criteria used to classify periodontal patients into stages and grades according to the 2018 WWP Classification System.
- Efforts should be made to report standard diagnostic accuracy and validity parameters, such as sensibility, specificity, negative and positive predictive values, compared to the reference standard, which should be correctly identified according to the case definition considered (e.g., furcation involvement, periodontitis).
- Future studies should evaluate the cost and time effectiveness of alternative and emerging imaging techniques introduced as complementary tools to the clinical assessment of periodontitis.
- The operator's learning curve necessary to reach proficiency in the implementation, use and interpretation of these alternative and emerging imaging techniques should be specifically evaluated in the field of periodontology.

## 5 Conclusion

Within the limitation of the present systematic review and meta‐analysis, and considering the moderate to high risk of bias of the included studies, the following conclusions can be drawn:

- Standard 2D radiography has high diagnostic accuracy in periodontitis assessment and can be considered as complementary to clinical diagnosis in the definition of the stage and grade.
- In the evaluation of furcation areas, the correlation between clinical measures and 2D radiography is highly heterogeneous with a tendency to underestimate the defects. Accuracy increases for more advanced level of involvement (i.e., Grades II and III furcation).
- CBCT is a precise, reliable and accurate tool, superior to intraoral radiographs, for the assessment of FI and intrabony defects. However, its use should be considered on a case‐by‐case basis by weighing the risk/benefit and cost/benefit ratios.
- Routine application of fractal analysis, USG and MRI will remain under validation until further studies highlight that their use for periodontitis diagnosis can be considered reliable, affordable, streamlined and with a positive cost/benefit ratio.

## Author Contributions

M.C.C. and N.D. contributed to the design and conception of the work, interpretation of the data and drafting and critically revising the manuscript. I.D.R. and C.W. contributed to data acquisition, analysis and interpretation and drafting of the manuscript. G.T. contributed to the acquisition and analysis of data and critically revised the manuscript. All authors contributed to and approved the final version of the manuscript to be published.

## Conflicts of Interest

The authors declare no conflicts of interest.

## Supporting information


**Figure S1.** Pooled data analysis for studies comparing CBCT versus clinical measurements as gold standard (PICO 2).

**Figure S2.** Publication bias observed for the diagnostic odds ratio (DOR) for PICO 1.

**Figure S3.** Publication bias observed for the diagnostic odds Ratio (DOR) for PICO 2.

**Figure S4.** Risk of bias and applicability concerns summary (PICO 1).

**Figure S5.** Risk of bias and applicability concerns summary (PICO 2).

## Data Availability

The data that support the findings of this study are available from the corresponding author upon reasonable request.

## Appendix 1

### Characteristics of Included Studies (PICO 1)

#### Patient‐Level Analysis (Table 1)

##### Study Design

The studies included in the patient‐level analysis comprised prospective cohort (Douglass et al. 1986) and cross‐sectional designs (Atchison et al. 1995; Farook et al. 2020; Machado et al. 2020; Walsh et al. 1997).

##### Disease Definition

The definition of periodontitis across the selected studies was heterogeneous. Investigations published after 2018 were based on the 2018 International Classification (Farook et al. 2020; Machado et al. 2020), while Walsh et al. (1997) used the definition based on the community periodontal index needs (CPITN) (Schürch et al. 1990). No precise periodontitis definition was provided across the other investigations: radiographic bone loss was the major parameter to define a ‘target’ patient (Table 1) (Atchison et al. 1995; Douglass et al. 1986).

###### Study Population/Selected Samples

Adult patients seeking a dental diagnosis were enrolled into two studies (Atchison et al. 1995; Douglass et al. 1986) and patients with an existing diagnosis of periodontitis in two other studies (Farook et al. 2020; Walsh et al. 1997). An epidemiological sample (SoPHiAS) was the source of Machado et al. (2020).

###### Reference Standard and Index Test

All selected studies used the clinical examination, including periodontal probing (UNC 15) as the reference standard against which to assess the diagnostic accuracy of the radiographic technique. Several combinations of radiographic techniques were evaluated. Panoramic radiography was evaluated in two studies (Machado et al. 2020; Walsh et al. 1997), bitewing and periapical radiographs in one study (Atchison et al. 1995), while both panoramic and periapical radiographs or their combination were evaluated by Farook et al. (2020) and Douglass et al. (1986).

#### Site‐Level Analysis (Table 2)

##### Study Design

A cross‐sectional study design was applied in six studies (Alasqah et al. 2022; Ashwinirani et al. 2015; Deas et al. 2006; Khocht et al. 1996; Kim et al. 2008; Saberi et al. 2017), while a longitudinal prospective study design was followed in four studies (Gedik et al. 2008; Hausmann et al. 1994; Rams et al. 1994). One publication was a retrospective study (Rams et al. 2018) and one a randomized clinical trial (Yusof et al. 2021).

##### Disease Definition

In accordance with the characteristics of the site analysed, a different specific classification or morphological description of the defect was applied. Furcation defects, classified according to the Hamp classification, morphological description of the marginal bone crest and the presence of radiographic angular bony defects were also taken into consideration.

##### Study Population/Selected Samples

Only one investigation used the 2018 International Classification definition of periodontitis (Yusof et al. 2021).

##### Reference Standard and Index Test

All investigations included used a clinical examination performed with a periodontal probe as the reference with which to compare the radiographic techniques.

Periapical radiographs were the most common radiological tool used to describe interproximal defects. Two publications used either only orthopantomograms (Saberi et al. 2017) or bitewing radiographs (Hausmann et al. 1994). The study by Ashwiniriani et al. (Ashwinirani et al. 2015) implemented the analysis with the use of radiovisiography, and the study by Gedik et al. (Gedik et al. 2008) analysed the defects with periapical, bitewing and panoramic radiographs. Only three publications specified whether the latter technique was deployed with any form of standardization.

### Characteristics of Included Studies (PICO 2)

Concerning the emerging imaging techniques and taking into account both the attempt to improve the diagnostic accuracy of the 2D examination and the attempt to reduce their biological radiation's effect, studies investigating the accuracy of CBCT are summarized in Table 3; those exploring the role of fractal analysis are summarized in Table 4; those exploring digital methods applied to analyse radiographic (ionising) imaging are shown in Table 5 and those exploring the diagnostic performance and application of non‐ionizing imaging methods in Table 6.

#### CBCT

##### Study Design

Overall, 29 studies investigated the diagnostic accuracy of CBCT; of these, one was a prospective cohort (Pajnigara et al. 2016) and one was an RCT (Yusof et al. 2021). The remaining 27 studies had a cross‐sectional design (Adurty et al. 2021; Alotaibi et al. 2024; Alsakr et al. 2022; Cimbaljevic et al. 2015; De Faria Vasconcelos et al. 2012; Fleiner et al. 2024; Goodarzi Pour et al. 2015; Grimard et al. 2009; Guo et al. 2016; Komšic et al. 2019; Lam et al. 2022; Li et al. 2015; Marinescu et al. 2014; Moradi Haghgoo et al. 2014; Nikolic‐Jakoba et al. 2021; Padmanabhan et al. 2017; Patil et al. 2023; Qi et al. 2023; Raichur et al. 2012; Suphanantachat et al. 2017; Walter et al. 2009; Yang et al. 2019; Zhang et al. 2018; Zhu and Ouyang 2016). The great majority (*n* = 26) were conducted in a university/dental college setting; only two (De Faria Vasconcelos et al. 2012; Goodarzi Pour et al. 2015) were conducted in private practice and one was not specified (Marinescu et al. 2014).

##### Disease Definition

The diagnostic accuracy of CBCT was assessed to detect and describe three types of periodontal defects: intrabony defects, furcation involvement and marginal bone crest (Table 3). Furcation involvement was most of the time diagnosed and classified based on Hamp's (Hamp et al. 1975) or Glickman's classification systems (Glickman and Carranza 1979). The number of defects analysed in the selected studies ranged between 20 and 200.

##### Study Population/Selected Samples

All studies included patients with periodontitis, history of periodontitis or seeking periodontal treatment. In some studies, the need of periodontal surgery was applied as a selection criterion.

##### Reference Standard and Index Test

Sixteen studies used intra‐surgical measurements as reference standard against which to evaluate CBCT accuracy (Banodkar et al. 2015; Goodarzi Pour et al. 2015; Grimard et al. 2009; Guo et al. 2016; Komšic et al. 2019; Li et al. 2015; Moradi Haghgoo et al. 2014; Nikolic‐Jakoba et al. 2021; Padmanabhan et al. 2017; Pajnigara et al. 2016; Patil et al. 2023; Qiao et al. 2014; Raichur et al. 2012; Walter et al. 2009; Yang et al. 2019; Yusof et al. 2021). The other studies used a clinical evaluation or peri‐apical radiographs as reference standard.

CBCT was the index text. The specific technical parameters used in the selected studies are detailed in Table 3.

#### Fractal Analysis

##### Study Design

Overall, 10 studies investigating the usefulness and accuracy of fractal analysis on periapical radiographs were included (Table 4) (Aktuna Belgin and Serindere 2020; Khajavi et al. 2017; Eser and Saribaş 2024; Korkmaz et al. 2023; Mishra et al. 2023; Sener et al. 2015; Shrout et al. 1998; Soltani et al. 2021; Updike and Nowzari 2008; Yarkac et al. 2023). All had cross‐sectional design and were conducted in a university‐based setting, except one that did not specify this aspect (Khajavi et al. 2017). Five studies were conducted in Turkey, two in the United States, two in Iran and one in India. All studies were designed to compare at least two groups of patients, such as patients presenting with periodontal health or gingivitis with patients with periodontitis or different stages of periodontitis (based on the 2018 International Classification definition of periodontitis).

##### Disease Definition and Study Populations

Heterogeneous clinical case definitions were applied, including the most recent ones based on the 2018 WWP Classification (Korkmaz et al. 2023; Mishra et al. 2023; Yarkac et al. 2023). Patients were selected from among those seeking periodontal treatment.

##### Reference Standard and Index Test

The reference standard was clinical examination in all studies.

The index test was the evaluation of the trabecular bone structure, most of the time assessed on first mandibular molars, based on the calculation of the fractal dimension (FD). This measure could be calculated on a periapical or panoramic radiograph by using the ImageJ software in all studies except one (Shrout et al. 1998).

#### Digital Methods to Analyse Radiographic Imaging and Non‐Ionizing Techniques

##### Study Design

There were nine prospective cohort studies, one controlled clinical trial, one case–control and one cross‐sectional investigation. These investigated computer‐assisted densitometric image analysis (CADIA) for alveolar bone density (Deas et al. 1991; Payne et al. 2013), digital subtraction analysis (Brägger et al. 1992; Eickholz and Hausmann 1997; Eickholz et al. 1998; Jeffcoat 1992; Preshaw et al. 1999; Reddy and Jeffcoat 1993), intraoral USG (Tanaka et al. 2023) and MRI (Juerchott et al. 2020; Probst et al. 2021). The study characteristics are summarized in Tables 5 and 6.

##### Disease Definition and Study Populations

All studies included patients with periodontitis (different case definitions) or seeking periodontal treatment. The target condition was the disease (i.e., periodontitis) in six studies, furcation involvement (FI) in four studies and intrabony defect or bone loss in two studies.

##### Reference Standard and Index Test

All studies used clinical examination or conventional radiography as the reference standard. In addition, Tanaka et al. (2023) and Juerchott et al. (2020), Juerchott et al. (2020) also compared the index technique with CBCT results (Tanaka et al. 2023; Juerchott et al. 2020).
