# Supplementary figures and images for: Accuracy of Ionizing‐Radiation‐Based and Non‐Ionizing Imaging Assessments for the Diagnosis of Periodontitis: Systematic Review and Meta‐Analysis
Source: J Clin Periodontol. 2025 Feb 12;52(Suppl 29):74–124. doi: 10.1111/jcpe.14137 (PMC12286653; doi:10.1111/jcpe.14137)

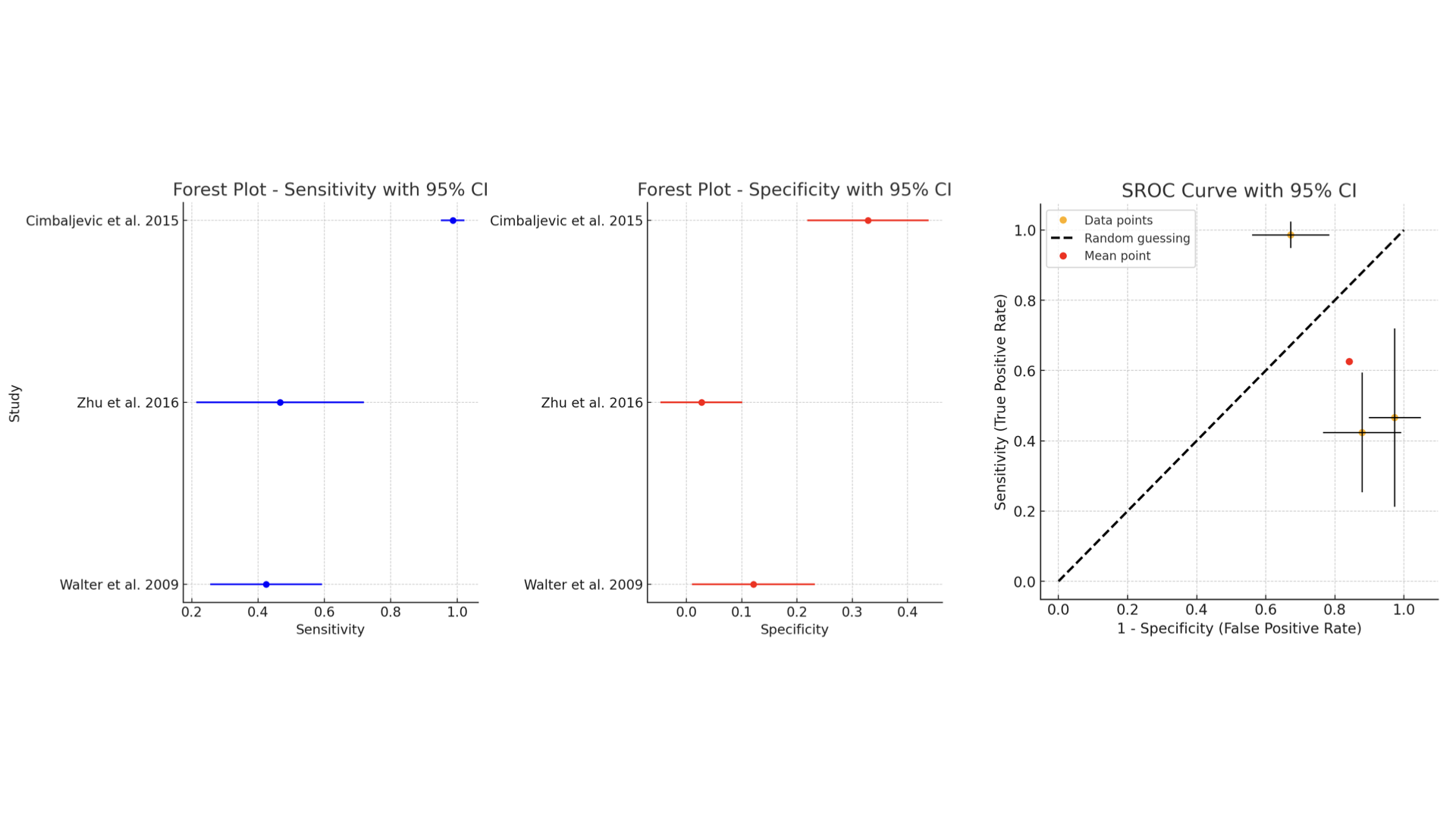

Supplement: Supplementary file 1 — Figure S1. Pooled data analysis for studies comparing CBCT versus clinical measurements as gold standard (PICO 2). [file JCPE-52-74-s005.tiff]

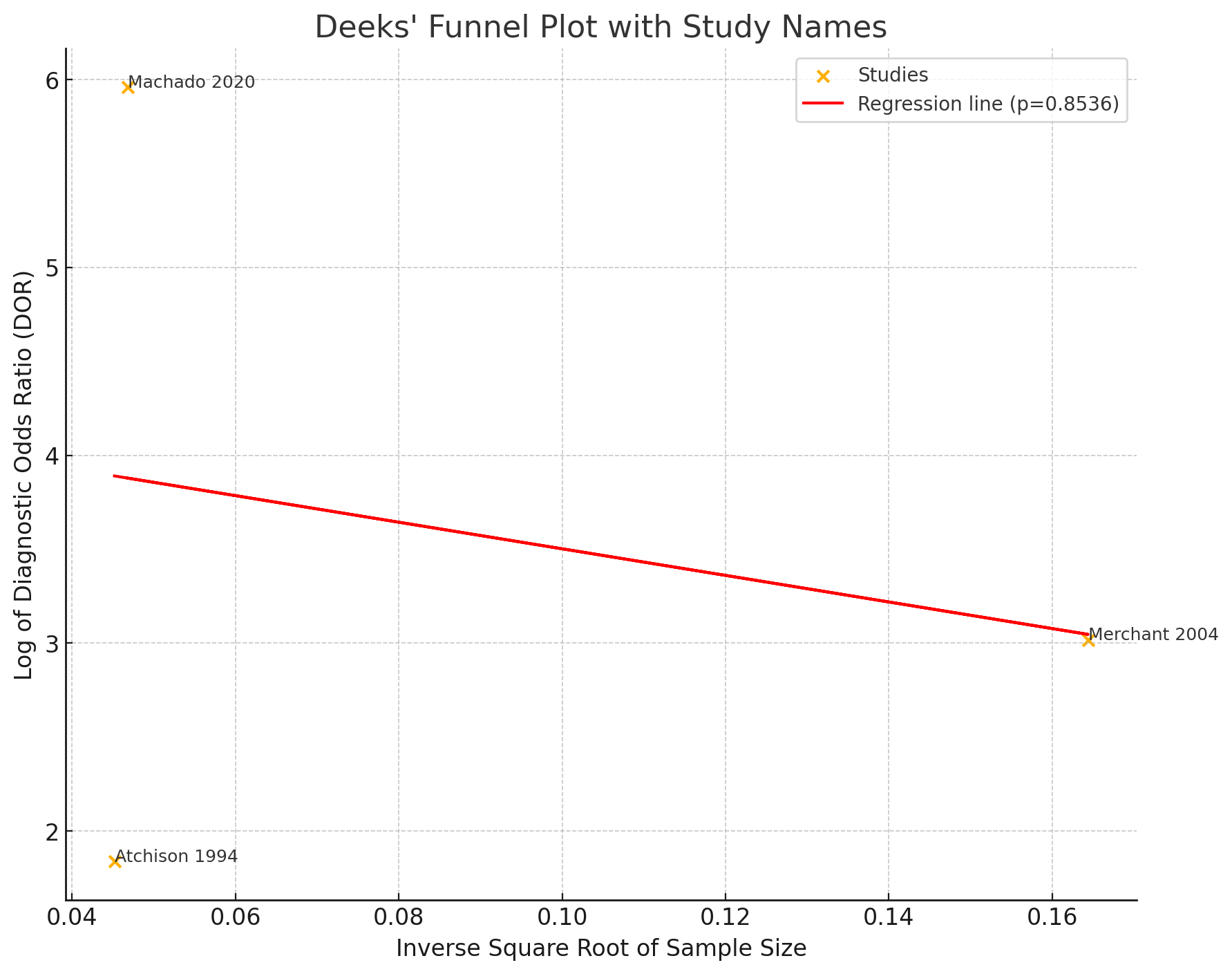

Supplement: Supplementary file 2 — Figure S2. Publication bias observed for the diagnostic odds ratio (DOR) for PICO 1. [file JCPE-52-74-s001.png]

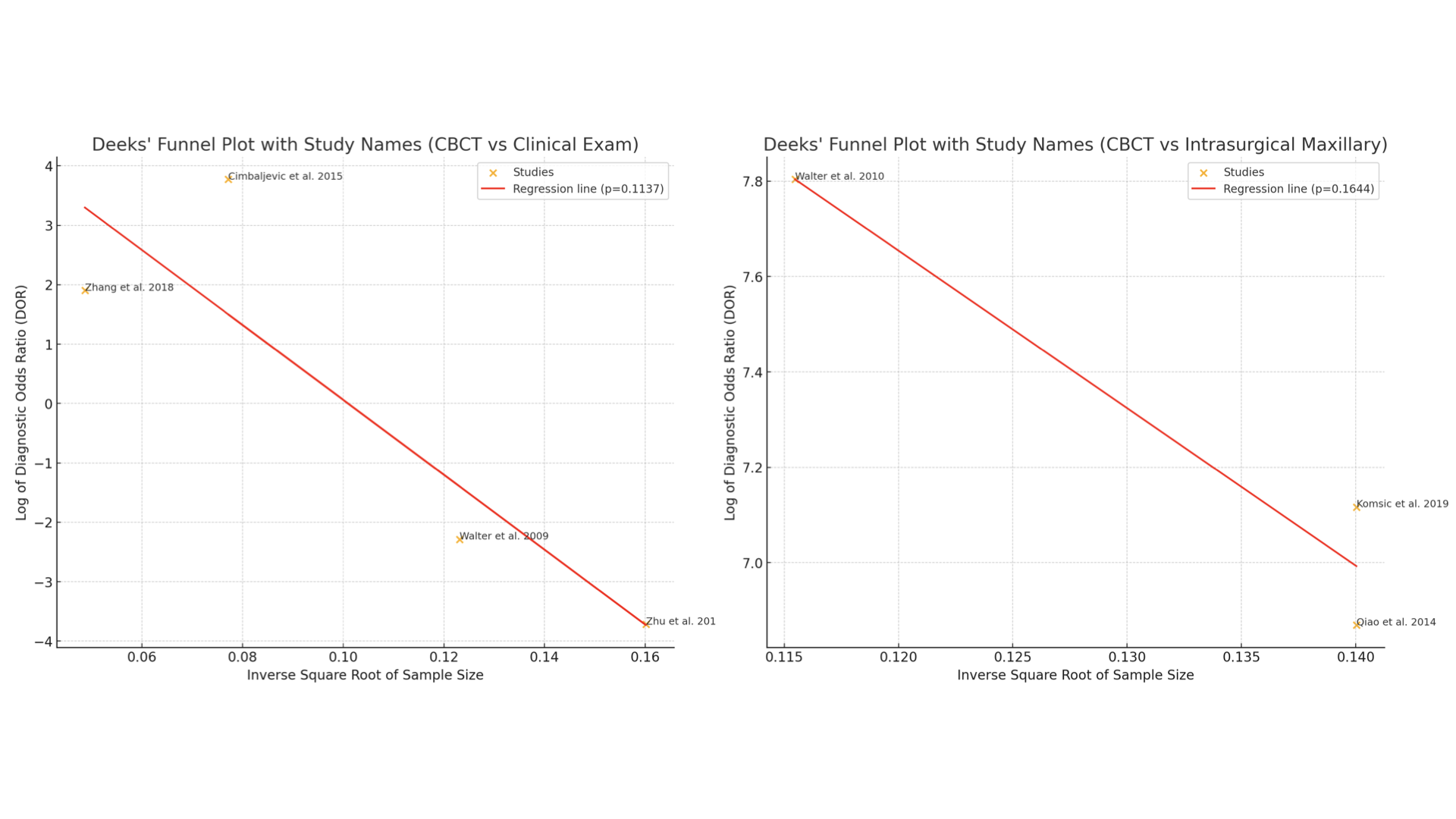

Supplement: Supplementary file 3 — Figure S3. Publication bias observed for the diagnostic odds Ratio (DOR) for PICO 2. [file JCPE-52-74-s004.tiff]
