# Supplementary material for: Accuracy of Ionizing‐Radiation‐Based and Non‐Ionizing Imaging Assessments for the Diagnosis of Periodontitis: Systematic Review and Meta‐Analysis
Source: J Clin Periodontol. 2025 Feb 12;52(Suppl 29):74–124. doi: 10.1111/jcpe.14137 (PMC12286653; doi:10.1111/jcpe.14137)
Supplement: Supplementary file 4 — Figure S4. Risk of bias and applicability concerns summary (PICO 1). [file JCPE-52-74-s002.docx]

**S. Figure 4. Risk of bias evaluation according to the QUADAS-II system of the study included based on PICO1 (n=26).**

| Risk of Bias | | | | |
| --- | --- | --- | --- | --- |
|  | | | | |
|  | Patient Selection | Index Test | Reference Standard | Flow and Timing |
| Douglass et al. (1986) |  |  |  |  |
| Papapanou et al. (1989) |  |  |  |  |
| Åkesson et al. (1992) |  |  |  |  |
| Hausmann et al. (1994) |  |  |  |  |
| Rams et al. (1994) |  |  |  |  |
| Atchison et al. (1995) |  |  |  |  |
| Khocht et al. (1996) |  |  |  |  |
| Machtei et al. (1997) |  |  |  |  |
| Walsh et al. (1997) |  |  |  |  |
| Eickolz et al. (1999) |  |  |  |  |
| Papelassi et al. (2000) |  |  |  |  |
| Zybutz et al. (2000) |  |  |  |  |
| Wolf et al. (2001) |  |  |  |  |
| Marchant et al. (2004) |  |  |  |  |
| Horr et al. (2005) |  |  |  |  |
| Deas et al. (2006) |  |  |  |  |
| Kim et al. (2008) |  |  |  |  |
| Gedik et al. (2008) |  |  |  |  |
| Ashwinirani et al. (2015) |  |  |  |  |
| Pahwa et al. (2015) |  |  |  |  |
| Saberi et al. (2017) |  |  |  |  |
| Rams et al. (2018) |  |  |  |  |
| Machado et al. (2020) |  |  |  |  |
| Farook et al. (2020) |  |  |  |  |
| Yusof et al. (2021) |  |  |  |  |
| Alasqah et al. (2022) |  |  |  |  |

| Applicability Concerns | | | |
| --- | --- | --- | --- |
|  | Patient Selection | Index Test | Reference Standard |
| Douglass et al. (1986) |  |  |  |
| Papapanou et al. (1989) |  |  |  |
| Åkesson et al. (1992) |  |  |  |
| Hausmann et al. (1994) |  |  |  |
| Rams et al. (1994) |  |  |  |
| Atchison et al. (1995) |  |  |  |
| Khocht et al. (1996) |  |  |  |
| Machtei et al. (1997) |  |  |  |
| Walsh et al. (1997) |  |  |  |
| Eickolz et al. (1999) |  |  |  |
| Papelassi et al. (2000) |  |  |  |
| Zybutz et al. (2000) |  |  |  |
| Wolf et al. (2001) |  |  |  |
| Marchant et al. (2004) |  |  |  |
| Horr et al. (2005) |  |  |  |
| Deas et al. (2006) |  |  |  |
| Kim et al. (2008) |  |  |  |
| Gedik et al. (2008) |  |  |  |
| Ashwinirani et al. (2015) |  |  |  |
| Pahwa et al. (2015) |  |  |  |
| Saberi et al. (2017) |  |  |  |
| Rams et al. (2018) |  |  |  |
| Machado et al. (2020) |  |  |  |
| Farook et al. (2020) |  |  |  |
| Yusof et al. (2021) |  |  |  |
| Alasqah et al. (2022) |  |  |  |
