# Supplementary material for: Accuracy of Ionizing‐Radiation‐Based and Non‐Ionizing Imaging Assessments for the Diagnosis of Periodontitis: Systematic Review and Meta‐Analysis
Source: J Clin Periodontol. 2025 Feb 12;52(Suppl 29):74–124. doi: 10.1111/jcpe.14137 (PMC12286653; doi:10.1111/jcpe.14137)
Supplement: Supplementary file 5 — Figure S5. Risk of bias and applicability concerns summary (PICO 2). [file JCPE-52-74-s003.docx]

**S. Figure 5. Risk of bias evaluation according to the QUADAS-II system of the study included based on PICO2 (n=51).**

| Risk of Bias | | | | |
| --- | --- | --- | --- | --- |
|  | Patient Selection | Index Test | Reference Standard | Flow and Timing |
| Deas et al (1991) |  |  |  |  |
| Brägger et al. (1992) |  |  |  |  |
| Jeffcoat et al. (1992) |  |  |  |  |
| Eickholz et al. (1997) |  |  |  |  |
| Shrout et al. (1998) |  |  |  |  |
| Eickholz et al. (1998) |  |  |  |  |
| Preshaw et al (1999) |  |  |  |  |
| Reddy et al. (2000) |  |  |  |  |
| Updike et al. (2008) |  |  |  |  |
| Grimard et al. (2009) |  |  |  |  |
| Walter et al. (2009) |  |  |  |  |
| Walter et al. (2010) |  |  |  |  |
| de Faria Vasconcelos et al. (2012) |  |  |  |  |
| Raichur et al. (2012) |  |  |  |  |
| Payne et al. (2013) |  |  |  |  |
| Haghgoo et al. (2014) |  |  |  |  |
| Marinescu et al. (2014) |  |  |  |  |
| Qiao et al. (2014) |  |  |  |  |
| Banodkar et al. (2015) |  |  |  |  |
| Cimbaljevic et al. (2015) |  |  |  |  |
| Goodarzi et al. (2015) |  |  |  |  |
| Li et al. (2015) |  |  |  |  |
| Sener et al. (2015) |  |  |  |  |
| Guo et al. (2016) |  |  |  |  |
| Pajnigara et al. (2016) |  |  |  |  |
| Zhu et al. (2016) |  |  |  |  |
| Pradmanabhan et al. (2017) |  |  |  |  |
| Suphanantachat et al. (2017) |  |  |  |  |
| Khajavi et al. (2017) |  |  |  |  |
| Zhang et al. (2018a) |  |  |  |  |
| Zhang et al. (2018b) |  |  |  |  |
| Komsic et al. (2019) |  |  |  |  |
| Yang et al. (2019) |  |  |  |  |
| Ruetters et al. (2019) |  |  |  |  |
| Aktuna Belgin & Serindere (2020) |  |  |  |  |
| Juerchott et al. (2020) |  |  |  |  |
| Adurti et al. (2021) |  |  |  |  |
| Nikolic-Jakoba et al. (2021) |  |  |  |  |
| Yusof et al. (2021) |  |  |  |  |
| Soltani et al. (2021) |  |  |  |  |
| Probst et al. (2021) |  |  |  |  |
| Alsakr et al. (2022) |  |  |  |  |
| Lam et al. (2022) |  |  |  |  |
| Patil et al. (2023) |  |  |  |  |
| Korkmaz et al. (2023) |  |  |  |  |
| Mishra et al. (2023) |  |  |  |  |
| Yarkac et al. (2023) |  |  |  |  |
| Tanaka et al. (2023) |  |  |  |  |
| Alotaibi et al. (2024) |  |  |  |  |
| Fleiner et al. (2024) |  |  |  |  |
| Eser & Saribas (2024) |  |  |  |  |

| Applicability Concerns | | | |
| --- | --- | --- | --- |
|  | Patient Selection | Index Test | Reference Standard |
| Deas et al (1991) |  |  |  |
| Brägger et al. (1992) |  |  |  |
| Jeffcoat et al. (1992) |  |  |  |
| Eickholz et al. (1997) |  |  |  |
| Shrout et al. (1998) |  |  |  |
| Eickholz et al. (1998) |  |  |  |
| Preshaw et al (1999) |  |  |  |
| Reddy et al. (2000) |  |  |  |
| Updike et al. (2008) |  |  |  |
| Grimard et al. (2009) |  |  |  |
| Walter et al. (2009) |  |  |  |
| Walter et al. (2010) |  |  |  |
| de Faria Vasconcelos et al. (2012) |  |  |  |
| Raichur et al. (2012) |  |  |  |
| Payne et al. (2013) |  |  |  |
| Haghgoo et al. (2014) |  |  |  |
| Marinescu et al. (2014) |  |  |  |
| Qiao et al. (2014) |  |  |  |
| Banodkar et al. (2015) |  |  |  |
| Cimbaljevic et al. (2015) |  |  |  |
| Goodarzi et al. (2015) |  |  |  |
| Li et al. (2015) |  |  |  |
| Sener et al. (2015) |  |  |  |
| Guo et al. (2016) |  |  |  |
| Pajnigara et al. (2016) |  |  |  |
| Zhu et al. (2016) |  |  |  |
| Pradmanabhan et al. (2017) |  |  |  |
| Suphanantachat et al. (2017) |  |  |  |
| Khajavi et al. (2017) |  |  |  |
| Zhang et al. (2018a) |  |  |  |
| Zhang et al. (2018b) |  |  |  |
| Komsic et al. (2019) |  |  |  |
| Yang et al. (2019) |  |  |  |
| Ruetters et al. (2019) |  |  |  |
| Aktuna Belgin & Serindere (2020) |  |  |  |
| Juerchott et al. (2020) |  |  |  |
| Adurti et al. (2021) |  |  |  |
| Nikolic-Jakoba et al. (2021) |  |  |  |
| Yusof et al. (2021) |  |  |  |
| Soltani et al. (2021) |  |  |  |
| Probst et al. (2021) |  |  |  |
| Alsakr et al. (2022) |  |  |  |
| Lam et al. (2022) |  |  |  |
| Patil et al. (2023) |  |  |  |
| Korkmaz et al. (2023) |  |  |  |
| Mishra et al. (2023) |  |  |  |
| Yarkac et al. (2023) |  |  |  |
| Tanaka et al. (2023) |  |  |  |
| Alotaibi et al. (2024) |  |  |  |
| Fleiner et al. (2024) |  |  |  |
| Eser & Saribas (2024) |  |  |  |
